# Reducing Nonradiative Losses in Perovskite LEDs through Atomic Layer Deposition of Al_2_O_3_ on the Hole-Injection Contact

**DOI:** 10.1021/acsnano.2c04786

**Published:** 2023-02-15

**Authors:** Emil G. Dyrvik, Jonathan H. Warby, Melissa M. McCarthy, Alexandra J. Ramadan, Karl-Augustin Zaininger, Andreas E. Lauritzen, Suhas Mahesh, Robert A. Taylor, Henry J. Snaith

**Affiliations:** † Clarendon Laboratory, Department of Physics, University of Oxford, Parks Road, Oxford, OX1 3PU, U.K.

**Keywords:** perovskite, light-emitting diode, atomic layer deposition, area selective, efficiency

## Abstract

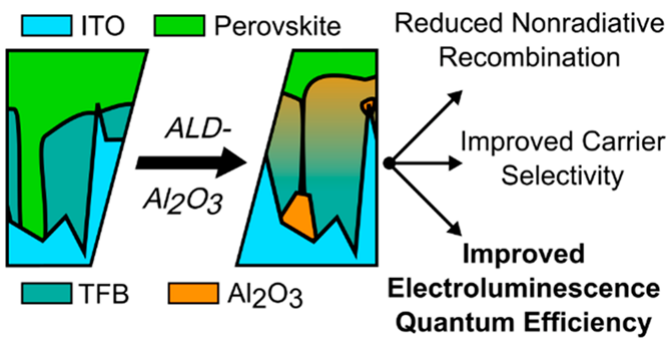

Halide perovskite light-emitting diodes (PeLEDs) exhibit great potential for use in next-generation display technologies. However, scale-up will be challenging due to the requirement of very thin transport layers for high efficiencies, which often present spatial inhomogeneities from improper wetting and drying during solution processing. Here, we show how a thin Al_2_O_3_ layer grown by atomic layer deposition can be used to preferentially cover regions of imperfect hole transport layer deposition and form an intermixed composite with the organic transport layer, allowing hole conduction and injection to persist through the organic hole transporter. This has the dual effect of reducing nonradiative recombination at the heterojunction and improving carrier selectivity, which we infer to be due to the inhibition of direct contact between the indium tin oxide and perovskite layers. We observe an immediate improvement in electroluminescent external quantum efficiency in our p-i-n LEDs from an average of 9.8% to 13.5%, with a champion efficiency of 15.0%. The technique uses industrially available equipment and can readily be scaled up to larger areas and incorporated in other applications such as thin-film photovoltaic cells.

## Introduction

Halide perovskite semiconductors have attracted enormous interest from the scientific community and industry over the past decade. A large variety of applications for this group of materials are being explored, including photovoltaic cells,^1^ radiation detectors,^2^ light-emitting diodes (LEDs),^3^ lasers,^4^ and photodetectors.^5^ Halide perovskites are interesting for LED applications, since they have highly efficient, tunable emission, with a narrow emission spectrum, and could feasibly outperform organic LEDs (OLEDs) in terms of absolute efficiency, due to the process of photon recycling.^6−9^ Since the report of the first room-temperature halide perovskite light-emitting diode (PeLED) in 2014,^3^ both publication numbers and efficiencies have soared. External quantum efficiencies of electroluminescence (EQE_EL_) above 20% have been reported by multiple groups since 2018.^10−13^ The rapid rise in efficiencies has been achieved through numerous improvements in design and fabrication, such as carrier confinement in the emitter,^14−16^ defect passivation strategies,^17−19^ improved carrier injection and transport,^20−22^ and thermal management strategies.^20,23^

PeLED devices are typically designed in both positive–intrinsic–negative (p-i-n) or negative–intrinsic–positive (n-i-p) heterostructure architectures with the emissive perovskite layer sandwiched between a hole transport layer (HTL) and an electron transport layer (ETL). An important property of the charge transport layer (CTL), and the contact that it forms in the device, is its “selectivity” for one carrier type, which can be defined as *S*_e_ = ρ_c*,*h_/(ρ_c*,*h_ + ρ_c*,*e_) = 1 – *S*_h_, where *S*_e_ is the electron selectivity of the contact, *S*_h_ is the hole selectivity, and ρ_c*,*h_ and ρ_c*,*e_ are the resistivities to hole and electron conduction through the contacts, respectively.^24^ It follows that a good CTL should be highly conductive for the majority carrier while being highly resistive for the minority carrier.

Organic HTLs in p-i-n device structures are typically deposited on transparent conducting oxides (TCOs) by spin-coating. Ideally, the CTL should provide full coverage of the TCO surface to reduce nonradiative recombination at the TCO–perovskite interface that would also cause a lack of selectivity of the contact due to a leakage of oppositely charged carriers from the emissive layer. Furthermore, the CTLs should be as thin as possible to reduce series resistance, which can cause loss due to Joule heating. In addition, the thicknesses of the HTL and ETL require optimization such that the hole and electron currents to the perovskite emitter match each other, facilitating efficient bimolecular recombination.^9^ The optical outcoupling efficiency (OOE) of the emitted light also depends on the refractive indices (*n*), extinction coefficients (*k*), and thicknesses of the constituent layers of the device. A device with a high internal electroluminescent quantum efficiency can have a relatively poor EQE_EL_ if the device stack (i.e., the perovskite and CTL thicknesses) is not optimized for a high outcoupling efficiency. It is clear to see that it is a significant challenge to balance all of these considerations to make high-efficiency devices.

Atomic layer deposition (ALD) is a layer-by-layer deposition technique which is surface controlled, allowing monolayer control of film growth.^25−27^ The ALD process consists of sequential, self-limiting surface reactions as alternating precursors and coreactants are introduced, separated by a purge of an inert gas. An ALD cycle may then be repeated to increase the number of layers and thus the thickness of the thin film deposited. Adequate reactive sites are required on the substrate surface for the precursor to react with, ensuring the self-limiting nature. If no reactive sites are available, no further deposition can take place. The uniformity and unparalleled conformality provided by this technique have led to its favored use in the scaling down of microelectronics.^25,28−30^ The low-temperature deposition and mild technique has also led to its application in perovskite solar cells^31−36^ as well as deposition on temperature-sensitive polymers.^37−42^

Area-selective ALD (AS-ALD) takes advantage of the strong dependence of ALD on surface chemistry, where the commencement of growth is conditional to the substrate surface.^43,44^ Here, deposition is limited to specific substrate areas, enabling predefined patterns to be established for bottom-up processing.^45−52^ Unreactive polymers^53−57^ as well as self-assembled monolayers (SAMs)^58−60^ have been used to allow surface modification and to mask selective reactive areas to prevent ALD growth. These materials act as a physical barrier, preventing the ALD precursor from reacting with substrate surface reactive groups. For unreactive polymers in this system, adequate ALD purges are required to inhibit the gaseous precursor from diffusing into the porous material and reaching the underlying substrate. If a sufficient purge is not applied, or if molecules become trapped in the porous polymer, these precursor molecules may then react with subsequent ALD precursors, leading to a delayed nucleation and eventual growth.^39,61^

In this work we develop a composite hole-injection layer composed of poly(9,9-dioctylfluorene-*alt*-*N*-(4-*sec*-butylphenyl)diphenylamine) (TFB) and Al_2_O_3_. We employ principles from AS-ALD to form an intermixed TFB–Al_2_O_3_ layer that blocks pinholes and thus inhibits nonradiative recombination sites in the interface between the perovskite and the hole-injection/electron-blocking layer. The result is an ultrathin transport layer with a very high resistivity to electrons, while efficiently injecting holes. Our champion LED efficiency improves from 12 to 15% EQE_EL_, confirming the improved properties of the composite hole-injection layer.

## Results and Discussion

For the green perovskite emitter, we employ the commonly used^15,16,20,62−65^ material CsPbBr_3_ within a phenylethylammonium bromide rich (PEABr) matrix, processed as we have previously reported.^64^ The emission wavelength varies depending upon the PEABr content. With 40 mol % excess PEABr in comparison to the CsBr, the wavelength sits within the green emission channel, with a PL peak position of 518 nm (Figure S1 in the Supporting Information).

**Figure 1:**
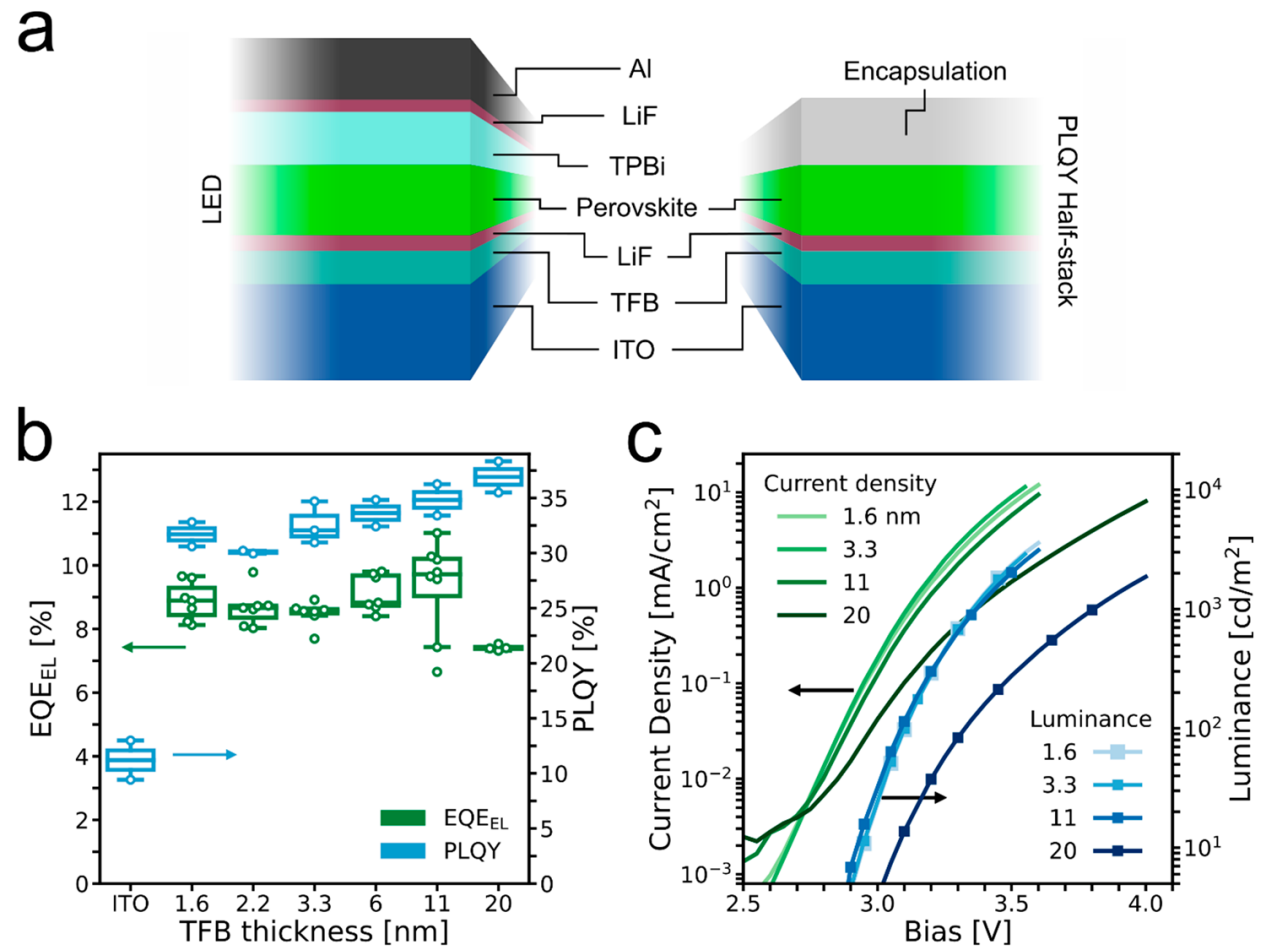
(a) Architecture of LED devices and half-stack samples for PLQY measurements. Thicknesses are not to scale. (b) Devices with varying TFB layer thicknesses and no Al_2_O_3_ interlayer. EQE_EL_ values are the highest recorded EQE_EL_ for each device, and PLQY values are the average value per sample. (c) *J*–*V*–*L* characteristics of representative devices with varying TFB layer thicknesses.

We integrate the emission layer in LEDs with a p-i-n architecture of ITO/poly(9,9-dioctylfluorene-*alt*-*N*-(4-*sec*-butylphenyl)diphenylamine) (TFB)/LiF/perovskite/2,2′,2″-(1,3,5-benzinetriyl)-tris(1-phenyl-1*H*-benzimidazole) (TPBi)/LiF/Al (Figure 1a). Using a similar structure, we have previously achieved an EQE_EL_ of up to 12% but observed a photoluminescence quantum yield (PLQY) of up to 28% from the same complete device stack.^64^ The discrepancy between EQE_EL_ and PLQY suggests there is an unrealized potential for higher efficiency in our devices. To improve the operating efficiency of the LEDs in this work, we adapt the HTL to optimize the hole-injection and electron-blocking nature of the p-side of the device.

To investigate how sensitive the LED performance is to the thickness of the hole-transport layer, we start by varying the thickness of the TFB layer by changing the concentration of TFB in the spin-coated solution and measure the PLQY on encapsulated “half-stacks” (ITO/HTL/LiF/perovskite) and EQE_EL_ of devices (Figure 1b). When the thickness of the TFB film is increased, the PLQY continues to increase, from an average of 32% for 1.6 nm TFB thickness to 37% at 20 nm thickness, whereas the EQE_EL_ drops off when the TFB thickness exceeds 11 nm. Samples with TFB films thinner than 11 nm all yield progressively lower PLQY and EQE_EL_, but even the thinnest TFB layer in conjunction with the LiF layer enables significantly higher PLQY than the perovskite on the bare ITO surface, with 32% versus 11% PLQY. The procedure for determining the thickness of the films is outlined in Note 1 in the Supporting Information.

Figure 1c shows the voltage dependence of the current density and luminance (*J*–*V*–*L*) of representative devices for each thickness. The device with the thickest TFB shows a strong reduction in both current density and luminance compared with those of the thinner ones. Among the three other devices there is almost no difference in luminance, but the current densities in the devices with the thinnest TFB films are slightly higher than that for the more efficient device with 11 nm TFB. The EQE_EL_ as a function of current density is given in Figure S3 in the Supporting Information.

A comparison of PLQY and EQE_EL_ provides valuable insights into the causes for emission losses, because PLQY does not rely on carrier transport and injection to deliver carriers to the emitter; thus, current transport losses and recombination losses can be uncoupled. In Figure 1b, we observe a general trend of increasing PLQY with increasing TFB thickness. This is consistent with the possible formation of “pinholes” in thinner TFB films. Contact between the perovskite emission layer with degenerately doped ITO would increase nonradiative recombination at the interface, leading to a reduction in emission, and, under bias, cause reduced selectivity, since electrons would be able to freely flow from the perovskite layer into the ITO. When the TFB thickness is increased, we assume the coverage over the ITO improves and reduced nonradiative recombination at this interface would lead to improvements in PLQY. If this is the case, there is a potential to improve the EQE_EL_ of devices with thinner TFB films compared to what we have achieved so far, by enabling improved coverage of the ITO. Notably, when we increase the TFB thickness above 11 nm, we observe a significant reduction in the injection current density under forward bias and a drop in the EQE_EL_. It is likely that this originates from either (a) an increased series resistance for hole injection, resulting in an imbalance of electrons and hole current flow through the device, or (b) a reduction in optical outcoupling, with thicker HTLs. An increase in series resistance for hole injection would only reduce EQE_EL_ and not PLQY, whereas a reduction in optical outcoupling should reduce both. We observe a monotonic increase in PLQY with increasing TFB layer thickness; however, we do acknowledge that our PLQY measurements are on half-stacks and could thus have a different outcoupling dependency on the thickness. Variations in thickness of this magnitude have been shown to result in changes in outcoupling efficiency.^66^ The concurrent reduction in current density with increased TFB thickness implies increased hole-injection resistance to be the primary factor reducing the EQE_EL_ for devices incorporating the thickest TFB layers.

### Identifying Nonradiative Recombination Pathways and Improving Carrier Selectivity

**Figure 2:**
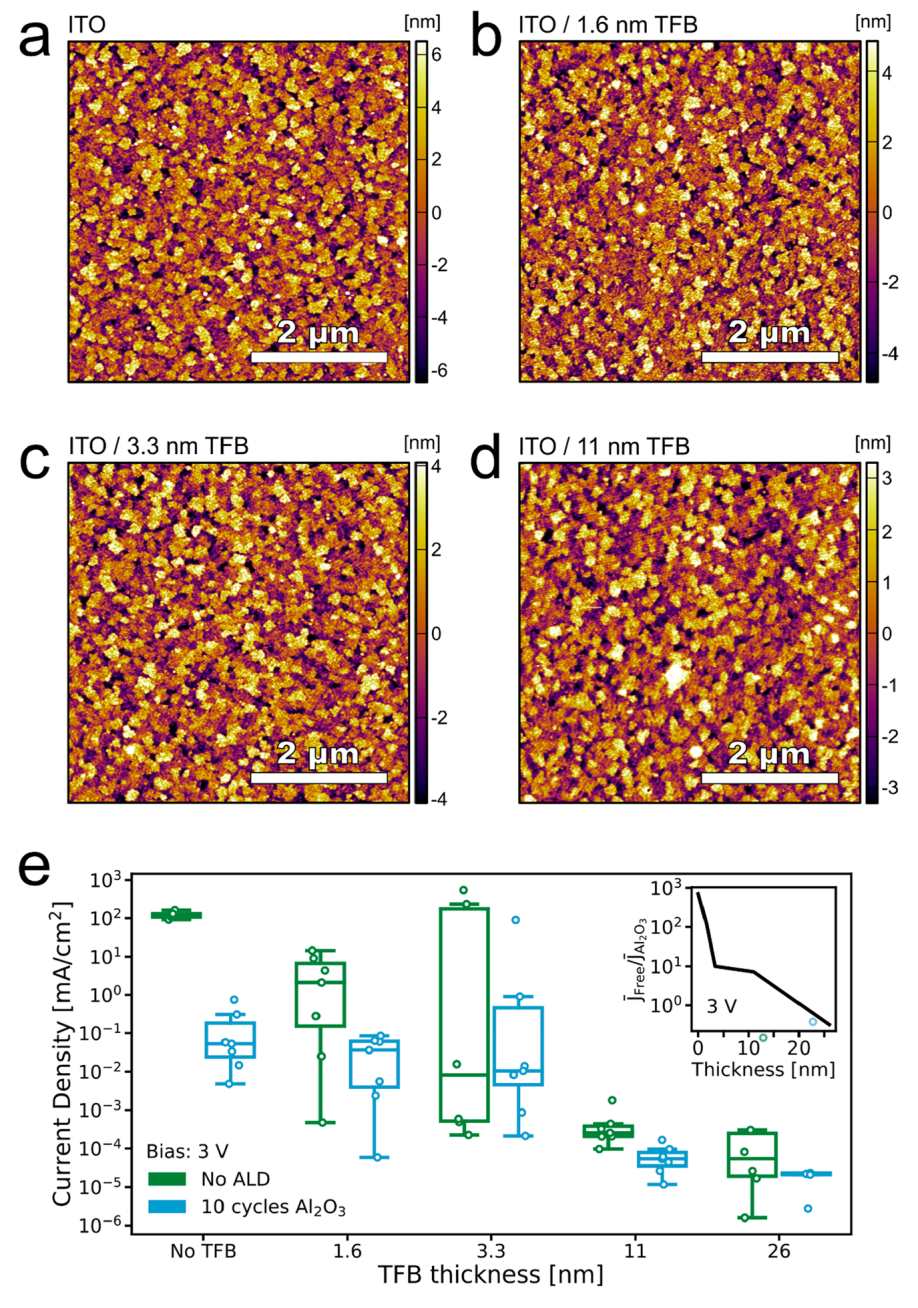
AFM micrographs of the topography of (a) the ITO surface, (b) ITO with 1.6 nm (0.5 mg/mL) TFB layer, (c) ITO with 3.3 nm (2 mg/mL) TFB layer, and (d) ITO with 11 nm (5 mg/mL) TFB layer. (e) Current density snapshot at 3 V bias for “electron-only” unipolar devices with varying TFB thicknesses and with or without the Al_2_O_3_ interlayer. In the inset in (e), the ratio of the mean of the current densities at 3 V for the Al_2_O_3_-free and Al_2_O_3_-covered samples is plotted as a function of TFB thickness.

To improve our understanding of the device performance, we investigate the morphology of the relevant layers in the device. We inspected the ITO and TFB layers by scanning electron microscopy (SEM) but were not able to discern any notable difference, possibly due to the penetration depth of the electron beam (Figure S4 in the Supporting Information). We then measured the topography using atomic force microscopy (AFM). Comparing scans of the ITO surface (Figure 2a) with scans of TFB thin films on ITO (Figure 2b–d), no clear difference can be discerned. The TFB thin films all display a topography similar to that of the bare ITO, suggesting a conformal deposition of the polymer. There is, however, a decrease in the root-mean-square roughness (RMS) of the TFB thin films with increasing film thickness (Table 1). The RMS roughness of the underlying ITO surface, at 3.3 nm, is in the same range as the thickness we measured of the thinner TFB layers employed. The reduction in roughness suggests that the TFB is partially planarizing the ITO substrate. Since the TFB thickness, for the thinner layers, is in the same range or less than the substrate roughness, it is likely that there will be some uncovered regions of ITO. Such uncovered regions protruding the TFB layer could act as contact points with the subsequently deposited perovskite emission layer.

**Table 1:** Root-Mean-Square Roughness of Surfaces in Figure 2a–d Determined by AFM

stack	roughness (nm)
ITO	3.3
ITO/1.6 nm TFB	2.7
ITO/3.3 nm TFB	2.3
ITO/11 nm TFB	1.7

With this potential for the presence of pinholes in the TFB layer, we suspect that the resulting ITO–perovskite interface that can form is increasing nonradiative recombination losses and decreasing the hole selectivity of the positive contact in our devices. To increase our understanding of how the TFB thickness may affect the selectivity of the positive contact, we construct unipolar, “electron-only” devices with the TFB layer sandwiched between two electron-selective contacts (Figure S5 in the Supporting Information).

In this configuration, the ITO/SnO_2_ contact should effectively block hole transport. This means the measured current flowing through the device should be due to electrons traveling through the entire device stack primarily, injected through the TPBi, passing through the SnO_2_, and collected at the ITO electrode. When sandwiching a hole-selective material such as TFB between the SnO_2_ and TPBi, we expect to see a reduction in measured current density due to the reduced electron transport through the thin-film stack. We do not expect to observe any hole injection from the ITO/SnO_2_ contact and hence only consider the electron current. TFB layers with incomplete coverage should still allow a substantial electron current because of the bare SnO_2_/TPBi interfaces. As such, this device configuration allows us to assess the completeness of the HTL layer coating in a more quantitative manner.

When we scan these unipolar devices (Figure S6 in the Supporting Information), we see a difference in current density of roughly 6 orders of magnitude between the thickest TFB layer and the devices without any TFB. Figure 2e shows the current densities of all devices at 3 V bias. The presence of 1.6 and 3.3 nm TFB layers decreases the current density by a few orders of magnitude but shows large variability between devices (green boxes in Figure 2e). For 11 nm and above the variability is smaller and the current density is significantly suppressed.

Having identified clear evidence for current leakage through the thinner TFB layers, we hypothesize that this can be inhibited by including an additional thin interlayer of an electrically insulating material. We proceed to compare with similar electron-only devices where we deposit a thin interlayer of Al_2_O_3_ via ALD on top of the TFB.

With an ALD-Al_2_O_3_ layer on top of the TFB in the sandwich between SnO_2_ and TPBi (blue boxes in Figure 2e), we see a further reduction in current density across all TFB concentrations. The relative reduction in electron current density due to the Al_2_O_3_ interlayer is of higher magnitude for the devices with no TFB, where the Al_2_O_3_ is deposited directly on the SnO_2_, and for the thinnest TFB layers.

In the inset in Figure 2e we plot the ratio of the mean of the current densities at 3 V for the Al_2_O_3_-free and Al_2_O_3_-processed samples (*J̅*_Free_/*J̅*_Al_2_O_3__). Without TFB, the mean current density is 700 times higher without the Al_2_O_3_ layer than with it. With the thinnest TFB layer, 1.6 nm, the ratio is reduced but remains high at 120. With 3.3 and 11 nm TFB, the Al_2_O_3_ interlayer reduces the electron current density by factors of 10 and 7, respectively. With a 26 nm TFB layer, the Al_2_O_3_ layer no longer causes a reduction in current density. In Note 2 in the Supporting Information, we inspect the morphology of the ITO/SnO_2_ contact and discuss the relevance of the data from the unipolar, electron-only devices compared to the ITO contact.

### Al_2_O_3_ Interlayer in LEDs

**Figure 3:**
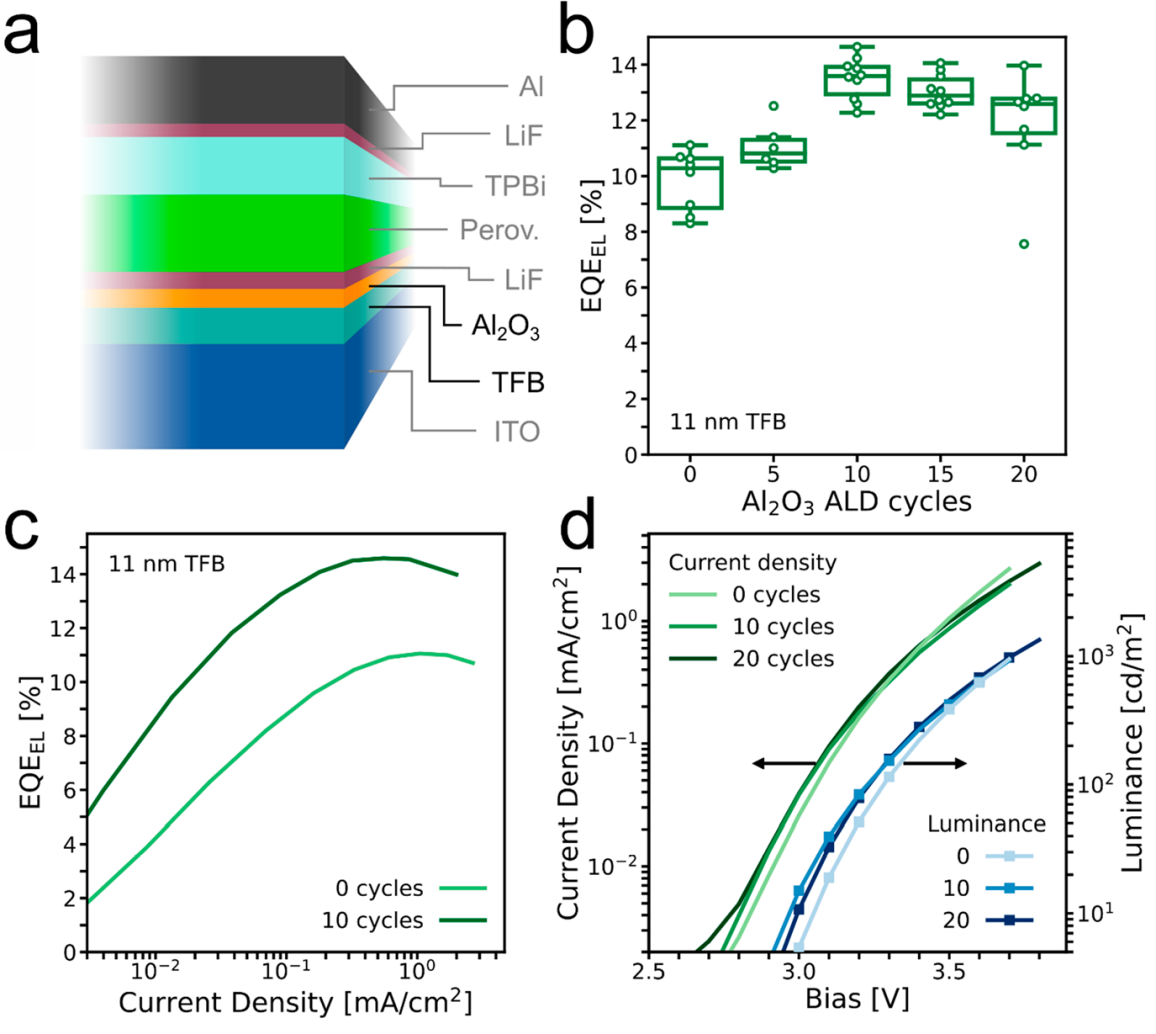
(a) Device schematic of the LEDs with the Al_2_O_3_ interlayer. (b) EQE_EL_ for each device with varying numbers of ALD cycles. (c) EQE_EL_ as a function of current density for the best-performing device with and without the interlayer from the batch in (b). (d) *J*–*V*–*L* characteristics of the best-performing devices with 0, 10, and 20 cycles from the batch in (b).

Having seen the reduction in electron leakage current with the ALD-Al_2_O_3_ interlayer in our unipolar devices, we now incorporate the interlayer into the full LED stack (Figure 3a). First, we make devices with varying numbers of deposition cycles of ALD, where one cycle involves an exposure to TMA and a subsequent exposure to deionized H_2_O vapor. We do this on our best-performing structure (11 nm TFB), and a clear increase in the EQE_EL_ with a maximum at 10 deposition cycles is observed (Figure 3b). The average EQE_EL_ rises from 9.8% at 0 cycles to 13.5% at 10 cycles.

The difference in EQE_EL_ between the 0 and 10 ALD cycle samples is significant over the entire current-density range measured (Figure 3c), yet the effect on the *J*–*V* curves is small (Figure 3d). This implies that the injected current and series resistance is not strongly influenced by the deposition of the Al_2_O_3_ interlayer, but the nonradiative current is significantly reduced. See also Note 3 in the Supporting Information.

Using AFM, we also measure the topography of samples of ITO/TFB/Al_2_O_3_. The effect of the ALD cannot be discerned from the micrographs, where samples with and without the interlayer look similar (Figure S9 in the Supporting Information). We also measure the perovskite layer but do not observe any changes in its morphology that can be attributed to the ALD-Al_2_O_3_ (Figure S10 in the Supporting Information).

**Figure 4:**
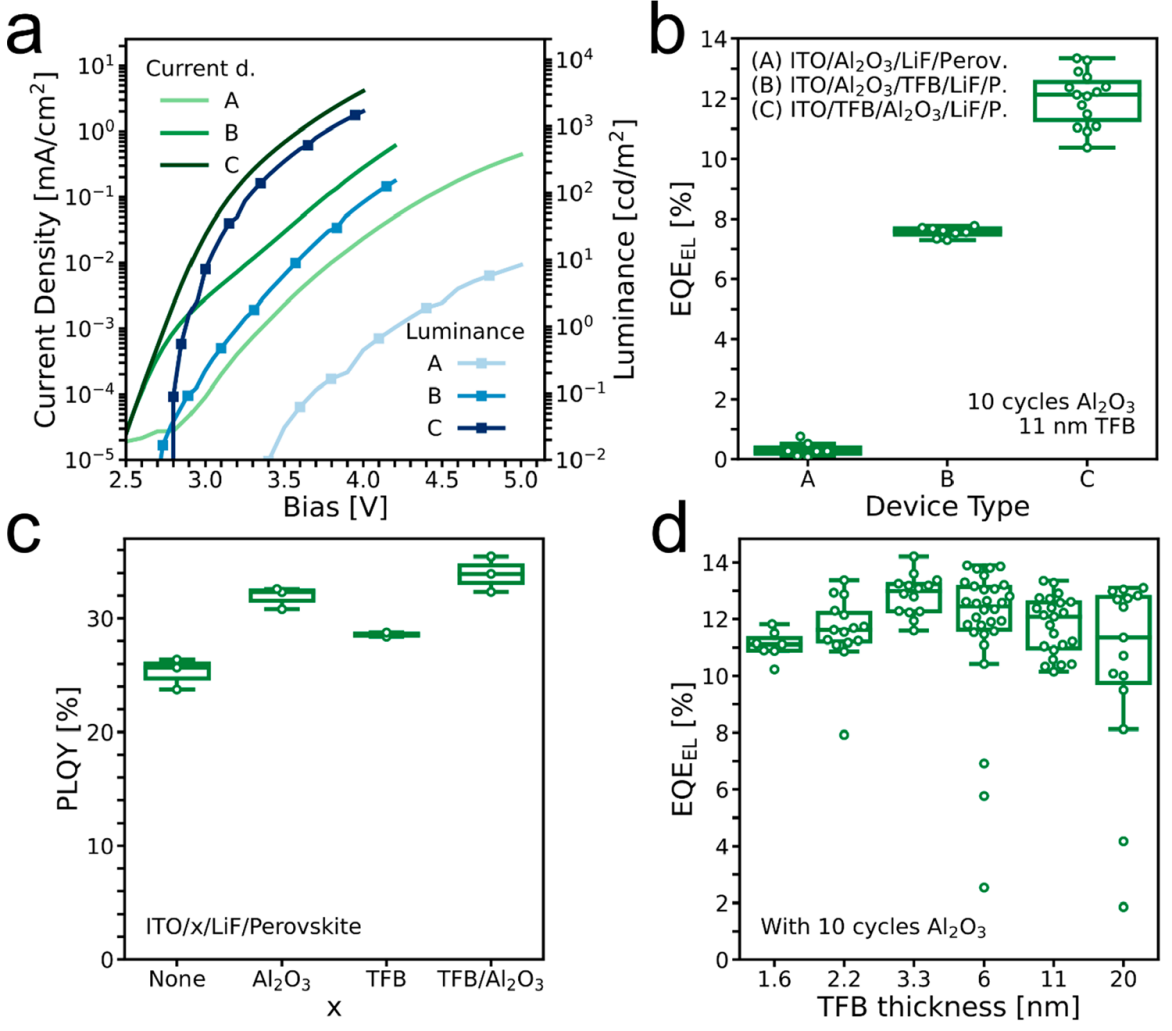
(a) *J*–*V*–*L* characteristics of different configurations of the 10 cycles of Al_2_O_3_ and 11 nm TFB layers: (i) no TFB HTL, only an Al_2_O_3_ interlayer; (ii) Al_2_O_3_ interlayer below the TFB; (iii) Al_2_O_3_ interlayer on top of the TFB. (b) EQE_EL_ of the same types of devices. (c) PLQY of devices with various layer configurations below the LiF wetting layer. (d) EQE_EL_ as a function of TFB thickness with the Al_2_O_3_ interlayer in place.

If the ALD-Al_2_O_3_ layer had formed a dense and continuous layer on top of the TFB, we would expect to see a considerable reduction in current density through the LED, but we do not observe this even at the highest number of cycles tested (Figure 3d). To further our understanding of the impact of this interlayer, we investigated two more configurations, removing the TFB layer and using only ITO/Al_2_O_3_/LiF as the hole-injection stack (type A) and inserting the Al_2_O_3_ interlayer under the TFB layer, i.e. depositing the oxide directly on the ITO before spin-coating the polymer ITO/Al_2_O_3_/TFB/LiF (type B), and compare this with the well-performing ITO/TFB/Al_2_O_3_/LiF hole-injection stack (type C) with 11 nm TFB and 10 cycles of ALD-Al_2_O_3_ (Figure 4a,b).

Without the TFB layer (type A), the LEDs have a very high resistance, the current density is reduced by 2 orders of magnitude relative to the control (type C), and the efficiency is below 1%. With the Al_2_O_3_ directly on the ITO and then TFB above (type B), the current density is increased but is still 1 order of magnitude below that of the control. The luminance of the type B devices is also much higher than that of type A devices but more than 1 order of magnitude lower than the control devices with TFB and then Al_2_O_3_ (type C). These results suggest that the Al_2_O_3_ deposition does not form a dense, predominantly continuous, insulating layer when it is applied on top of TFB but does form a more continuous and insulating layer on ITO. Thus, our original configuration still allows efficient hole injection through the TFB to the perovskite emission layer.

We now inspect the effect of 10 cycles of the aluminum oxide interlayer on the PLQY of half-stacks, as shown in Figure 4c, and find an increase of 5% absolute PLQY with the ALD-Al_2_O_3_ layer processed on top of the 11 nm TFB layer. We also see that the combination of the thin Al_2_O_3_ and LiF interlayers is able to quite effectively suppress nonradiative recombination at the ITO interface, resulting in high PLQYs.

Now that we have found an effective method to improve our contacts by reducing nonradiative recombination, we try to optimize the LEDs further by varying the TFB layer thickness while keeping the ALD-Al_2_O_3_ layer constant at 10 cycles. As shown in Figure 4d, the optimum TFB thickness is shifted such that we now find the highest efficiencies in devices prepared with 3.3 nm TFB layers. The champion EQE_EL_ in Figure 4d is 14.2%, but we note that we have reached 15.0% in a 11 nm device with an Al_2_O_3_ interlayer early in this study (Figure S11 in the Supporting Information) and we therefore expect to be able to obtain even higher efficiencies for this configuration with 3.3 nm TFB and 10 cycles of ALD-Al_2_O_3_. We note that even the devices with 20 nm TFB show a significant improvement in EQE_EL_ with the Al_2_O_3_ interlayer (comparing the data presented in Figure 1b and Figure 4d). JVL and EQE_EL_(*J*) for the different concentrations with the Al_2_O_3_ interlayer are given in Figures S12 and S13 in the Supporting Information, respectively. The LED operational stability over time remains largely unchanged by the inclusion of the Al_2_O_3_ interlayer (Figure S14 in the Supporting Information).

We thus see an improvement in device performance with the Al_2_O_3_ interlayer across all TFB thicknesses, with a greater change in samples where the TFB coverage is expected to be lower because of the lower thickness. We can attribute this to the difference in growth characteristics of the Al_2_O_3_ on ITO and on TFB.

In the ALD process of Al_2_O_3_, the trimethylaluminum precursor reacts with functional groups on the surface to chemisorb on the substrate. In the subsequent steps of the cycle, the gaseous TMA is purged out of the deposition chamber and the second precursor, in this case H_2_O, is let into the chamber. The H_2_O reacts with the chemisorbed TMA to form a monolayer of aluminum oxide. The nucleation of the Al_2_O_3_ layer is therefore strongly dependent on the chemistry between the TMA precursor and the substrate surface. As metal oxides, the surfaces of both ITO and SnO_2_ are likely to be terminated by hydroxide groups, which react strongly with TMA. Therefore, a short and effective nucleation phase is expected on these surfaces before the process enters the linear growth phase that is characteristic for Al_2_O_3_ ALD growth.^43^

On the other hand, many polymers do not possess a high density of functional groups for TMA to bind with and are relatively inert to the ALD nucleation process.^61^ Inspecting the molecular structure of TFB (Figure S15 in the Supporting Information), it is clear that there are no −OH, −COOH, or primary amine groups for TMA to react with. TMA, being a strong Lewis acid, could in theory react with the amine group (Lewis base) on the TFB monomer. However, since this is a tertiary amine, it is not expected to be very reactive.

If TMA cannot readily react on the TFB surface, another possibility is for the gaseous TMA molecules to diffuse into micropores within the polymer layer. If, during the following purge, a fraction of the TMA molecules is unable to escape the pores of the TFB, the TMA will then be free to react with H_2_O molecules in the next pulse. This would initiate a nucleation and growth of Al_2_O_3_ particles within the TFB film, effectively resulting in a composite of a TFB matrix infused with Al_2_O_3_ particles.

**Figure 5:**
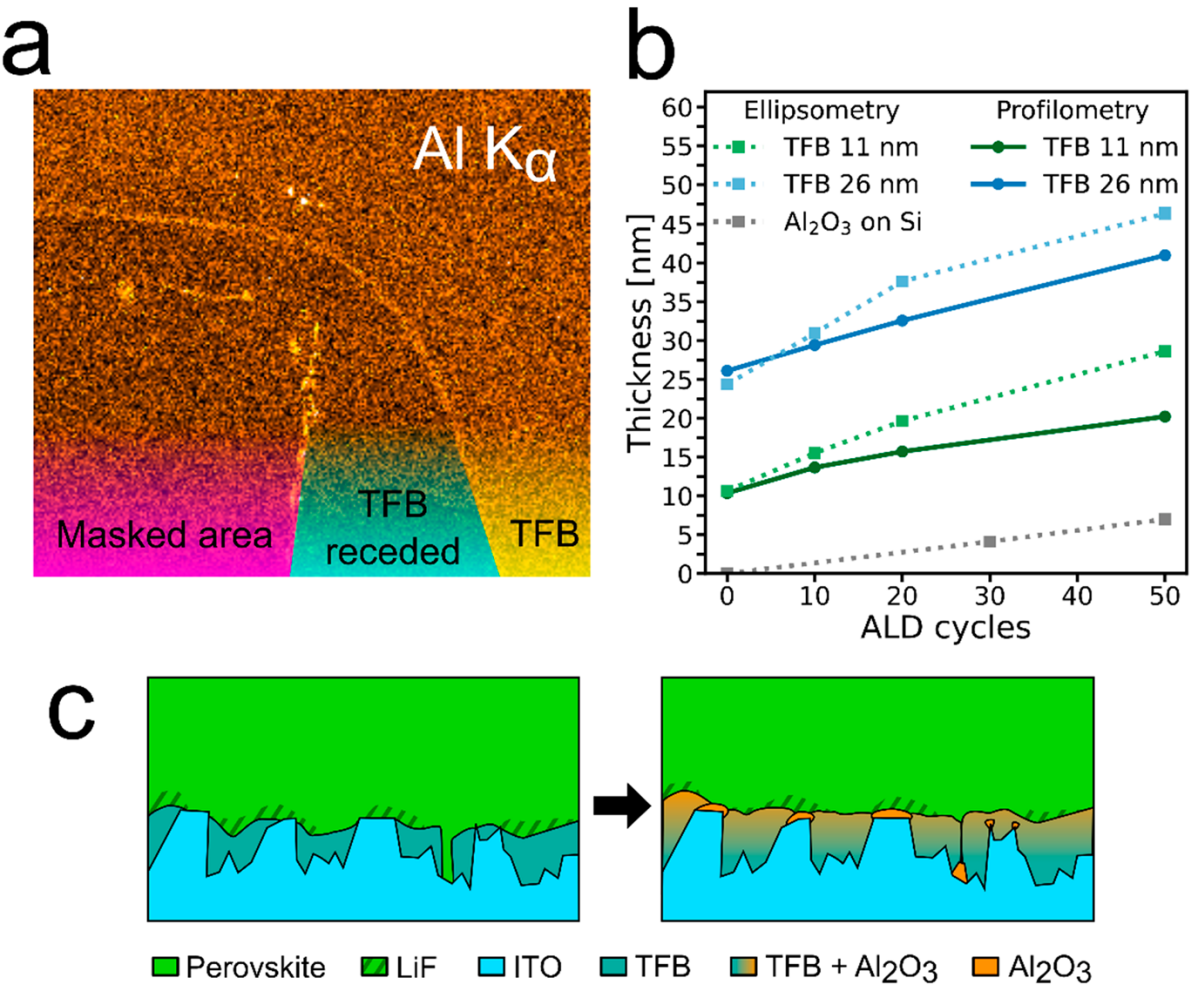
(a) Al Kα counts from EDS mapping over a sample with 11 nm TFB on ITO and then 20 cycles of ALD-Al_2_O_3_, where a part of the sample was masked by polyimide tape during TFB spin-coating. An increased brightness indicates a higher count intensity. The color coding indicates: (magenta) the area that was masked and is therefore TFB-free, (cyan) an area around the masking tape where the TFB had receded after spin-coating, and (yellow) a TFB-covered area. (b) Thicknesses of TFB-Al_2_O_3_ samples on Si and Al_2_O_3_ on Si samples as a function of ALD-Al_2_O_3_ cycles, where the thicknesses were determined by profilometry (average values) and ellipsometry. (c) Illustration of ALD-Al_2_O_3_ causing a swelling of the TFB by a,intermixed growth of Al_2_O_3_, and growth of Al_2_O_3_ on exposed ITO surfaces. The drawing is not to scale.

To gain an understanding of the growth of the Al_2_O_3_ interlayer, we performed energy dispersive X-ray spectroscopy (EDS) mapping in SEM on a sample of ITO with 11 nm TFB and 20 cycles of ALD-Al_2_O_3_, where we masked parts of the substrate with polyimide tape during the TFB spin-coating and then removed the tape to expose this area during ALD. The data are shown in Figure 5a and Figure S16 in the Supporting Information. We see an increased intensity of Al Kα counts in the area with TFB coverage compared to that in areas with just ITO. This implies that there is a larger amount of Al_2_O_3_ in this area. This suggests we may have some Al_2_O_3_ growth in the TFB pores.

To further investigate the potential intergrowth of Al_2_O_3_ within TFB, we deposit TFB and TFB–Al_2_O_3_ layers on silicon substrates and measure the thickness using both profilometry and ellipsometry (see Figure 5b and Figure S17 in the Supporting Information). We see from Figure S17a,b in the Supporting Information that the thicknesses determined by the two different methods follow each other closely, except at higher ALD cycles the thickness determined by ellipsometry is consistently slightly higher than the average values given by profilometry. Figure S17c in the Supporting Information shows the increase in thickness as a function of ALD cycles relative to the bare TFB (0 cycles). The measured thickness increase exceeds the value expected from linear growth of Al_2_O_3_ by ALD (1.1 Å per cycle),^43^ which would be expected from a continuous film on the TFB surface. In contrast, our deposition of Al_2_O_3_ directly on a Si substrate with native oxide follows this rate much more closely (Figure S17b,c in the Supporting Information). After 10 cycles, the 11 nm TFB-Al_2_O_3_ film increases in thickness by 3–5 nm, or 30–50%, relative to the pristine TFB film (Figure S17d in the Supporting Information), much more than the expected 1.1 nm. The layer continues to expand substantially also for 20 and 50 cycles of ALD, and the trend is similar for a thicker pristine TFB film of 26 nm.

From these data, we draw the conclusion that the Al_2_O_3_ likely grows within pores in the TFB film, resulting in swelling of the TFB layer and the formation of a TFB–Al_2_O_3_ composite layer. The number of deposition cycles explored in devices in this work (0–20 cycles) is also well within the range considered to be the nucleation phase for growth on many common polymers, before which a conformal linear growth is reached.^39^ This could further help explain why there does not seem to be a conformal, insulating blocking layer of Al_2_O_3_ formed, despite there being a significant volume of Al_2_O_3_ within the film.

We thus propose that the application of ALD-Al_2_O_3_ acts by improving the devices in two ways: (1) any uncovered ITO regions on the substrates may be insulated by a more reactive growth in this area, forming a thin but dense and conformal film, and (2) the surface of the TFB is not insulated but the swelling could also act to seal micropores and cause a more continuous surface for subsequent layers in the device fabrication. See the schematic in Figure 5c.

This premise is consistent with the changes we observed for the different LED device configurations. For our “control” devices, incomplete coverage or pinholes in the TFB film lead to nonradiative electron–hole recombination and poor selectivity at the perovskite interface with the ITO (Figure S18a in the Supporting Information). When applying the ALD-Al_2_O_3_ process, hole conduction through the TFB is sustained, since the Al_2_O_3_ does not form a dense, continuous layer capping the TFB but rather interpenetrates the polymer. This therefore greatly reduces the electron leakage current, while allowing hole-injection current to flow (Figure S18b,c in the Supporting Information). This confines a higher proportion of the injected electrons to the emitter layer, where they can radiatively recombine with the holes. With the TFB-Al_2_O_3_ HTL, reduction of the initial TFB thickness, even accounting for swelling post ALD, reduces the hole injection resistance, without the drawback of increased nonradiative recombination from the increasingly large area of exposed ITO surface.

We see an EQE_EL_ increase with 10 cycles of ALD-Al_2_O_3_ also for the thicker TFB layers (Figure 4d), which is indicative that there could be other positive effects associated with the TFB–Al_2_O_3_ composite. The composite could have a better energetic alignment compared to that of neat TFB, facilitating improved hole injection into the perovskite layer. Another possibility is that the composite aids in enhancement of the following layer depositions, either the evaporated LiF or the spin-coated perovskite layer. However, our experience with this emission layer indicates that the perovskite crystallization is mostly governed by the 18-crown-6 additive and the presence of the LiF wetting layer.^64^ It could also be the case that the reduced HTL thickness has improved the optical outcoupling of the device, though over the layer thickness range (between 3 and 11 nm), which is significantly less than the wavelength of light, we would not expect dramatic changes. A thorough optical model would be needed to confirm this, which is beyond the scope of this work.

This technique, exploiting differences in chemical reactivity of ALD precursors and growth modes between the organic HTL and the underlying TCO to achieve selective insulation of leakage pathways, thus widens the optimization window for device design by expanding the range of viable organic charge transport layer thicknesses that can be used to produce highly efficient devices. In our work, the efficiency gain primarily comes from improved selectivity of the contact. However, in other applications the technique also offers the possibility to optimize the optical outcoupling efficiency through variations of layer thickness without losing carrier selectivity. The combination of an insulator by ALD with an organic charge transport layer may in the future be adapted to other organic charge transport layers, other device architectures, such as n-i-p devices, or other fields such as perovskite photovoltaics. Since ALD systems are common in semiconductor research laboratories, the technique has potential for fast implementation into a large variety of applications. Through commercially available spatial and roll-to-roll ALD systems, the technique should be suitable for scale-up to larger-area LED arrays.^67^

## Conclusion

The imperfect wetting associated with solution processing can introduce serious spatial inhomogeneities in PeLEDs, presenting a major challenge to scale-up. This challenge is particularly exacerbated by the requirement to have very thin transport layers for the highest efficiencies. Here, we show how ALD can be exploited to improve the quality of the hole-injection layer and contacts, which we rationalize to be due to preferential blocking of regions with pinholes through both swelling of the polymer layer caused by Al_2_O_3_ growth in the pores of the polymer and preferential Al_2_O_3_ growth on exposed TCO surfaces, while the polymer surface remains electronically accessible to the perovskite emitter. The improved TFB–Al_2_O_3_ contact reduces nonradiative recombination sites where there is contact between the degenerately doped ITO and the perovskite emission layer, improving the selectivity of the contact. This allows us to improve the hole injection into the emission layer further by reducing the thickness of the HTL, thus reducing the series resistance for hole injection without introducing additional nonradiative recombination sites. The technique is broadly applicable to a variety of organic transport layers and could expand the optimization window of a variety of devices architectures without a tradeoff in performance due to loss in contact selectivity.

## Methods

### Materials

Lead(II) bromide 99.999% (35703, Alfa Aesar), cesium bromide 99.999% (429392, Sigma-Aldrich), 18-crown-6 ≥99.0% (274984, Sigma-Aldrich), TFB (poly(9,9-dioctylfluorene-*alt*-*N*-(4-*sec*-butylphenyl)diphenylamine)), AD259BE, American Dye Source, Inc.), TPBi (2,2′,2″- (1,3,5-benzinetriyl)-tris(1-phenyl-1*H*-benzimadizole, LT-E302, Lumtec Inc.), tin(IV) oxide (44592, Alfa Aesar), LiF (LT-E001, Lumtec Inc.), phenylethylamine (128945, Sigma-Aldrich), ethanol (443611, Sigma-Aldrich), dimethyl sulfoxide (276855, Sigma-Aldrich), chlorobenzene (284513, Sigma-Aldrich), and trimethylaluminum (93–1360, Strem) were all used as purchased without further purification.

PEABr was prepared in-house for a previous project; see Warby et al.^64^

### Perovskite Film Fabrication

Perovskite precursor solutions were prepared by weighing out precursor salts in a molar ratio of 5:5:2 of PbBr_2_:CsBr:PEABr directly into a single vial. DMSO was placed in the vial to a volume corresponding to the PbBr_2_ salt being at a 0.2 M concentration. The crown ether 18-crown-6 was included in the precursor solution as a structuring agent at a concentration of 4 mg/mL. The solutions were stirred overnight at room temperature and then filtered with a 0.45 μm PTFE membrane filter. Perovskite films were fabricated by spin-coating. The spin-coating program consisted of a first step of 5 s duration at 1000 rpm (1000 rpm/s ramp) followed by a second step of 60 s duration at 3000 rpm (3000 rpm/s ramp). A 100 μL portion of the solution was dispensed onto the spinning substrate 1–2 s into the first step. After the spin-coating procedure the substrates were immediately moved to a hot plate and annealed for 10 min at 100 °C. All steps took place in a N_2_-filled glovebox.

### LED Fabrication

Glass substrates with a patterned layer of ITO (*R*_s_ ≤ 10 Ω) from Shenzhen Huayu Union technology Co. Ltd. were cleaned by scrubbing with a toothbrush in a solution of deionized (DI) water and Decon 90 detergent. The substrates were then sonicated for a minimum of 3 min per step in the detergent solution, deionized (DI) water, acetone, and isopropanol and then dried using a N_2_ gun. Before the deposition of any layers, the cleaned ITO substrates were exposed to a 10 min UV–ozone cleaning process.

TFB solutions were prepared by weighing out TFB powder in a vial and adding chlorobenzene to obtain the desired concentration. The solutions were stirred overnight at 70 °C and then filtered with a 0.22 μm PTFE filter. When testing multiple concentrations, a solution of the highest concentration was made and then diluted into separate vials after filtering to obtain a spectrum of concentrations. TFB layers were spin-coated onto the ITO substrates in ambient air at 4000 rpm for 30 s and then annealed at 120 °C for 10 min.

A homemade ALD system was used to deposit Al_2_O_3_ at 100 °C. In each cycle, TMA was introduced with a residence time of 5 s before purging for 30 s. DI H_2_O vapor was subsequently introduced with a residence time of 5 s and a following purge for 40 s. N_2_ was used as both a carrier and purge gas for the process.

LiF was deposited by thermal evaporation in a vacuum chamber positioned in a N_2_ glovebox. 2 nm was evaporated for the wetting layer below the perovskite, at a rate of 0.1 Å/s. From this step onward, the samples were kept in an inert atmosphere at all times to prevent reactions with ambient water or oxygen. Perovskite was deposited on LiF-covered substrates by spin-coating as described above.

The samples were again transferred to the thermal evaporator, and a 45 nm layer of TPBi at a rate of 1 Å/s and a 1 nm layer of LiF were evaporated. Then, 80 nm of Al was evaporated at a rate of 1 Å/s using an evaporation mask defining the device layout.

### Unipolar Device Fabrication

Unipolar, electron-only devices were fabricated using the same substrates and substrate cleaning procedure as for the LEDs. A 2.67% SnO_2_ solution was made by diluting the 15% colloidal dispersion with DI water. The SnO_2_ layer was spin-coated onto the ITO substrates using a 30 s spin program at 3000 rpm and dispensing 50 μL of the solution 2 s into the program. The samples were then annealed for 30 min at 150 °C. Before spin-coating TFB on top of the SnO_2_, the samples were UV–ozone cleaned for another 10 min to improve wetting. The TFB, Al_2_O_3_, TPBi, LiF, and Al layers were deposited as described for the LED fabrication.

### Sample Fabrication for PLQY

Samples for PLQY measurements were prepared in the same way as for the LEDs for all layers up to the emitter. On the ITO-only sample the perovskite was spun onto the UV–ozone cleaned ITO without a LiF wetting layer. All other layers were deposited as described above. The samples were encapsulated with glass slides bonded on top of the perovskite emitter by use of a Dymax OP-29 UV curing glue, cured for 2 min. The glue was dispensed by a preprogrammed robot, ensuring an optimal dispensing amount and minimal flow into the center of the sample, leaving a large area of pristine perovskite for the PLQY measurements.

### Sample Fabrication for Ellipsometry, XRR, and Profilometry

Samples for determination of TFB, TFB-Al_2_O_3_, and SnO_2_ film thicknesses were made on various substrates: ITO, Si, and glass. In each case, the substrate was wet-cleaned as described above for LED fabrication. The substrates were then UV–ozone cleaned (ITO, Si) or plasma cleaned (glass) for 10 min directly prior to spin-coating TFB or SnO_2_. TFB or SnO_2_ was spun with the same parameters as described for LED fabrication and unipolar device fabrication.

### Sample Fabrication for AFM, SEM, and EDS

Samples for AFM and SEM with ITO substrates were prepared following the exact same steps as described for LED devices. Glass substrate samples for AFM were prepared as described above for ellipsometry, XRR, and profilometry. For EDS samples with masking, a polyimide tape was placed across parts of the ITO substrate before TFB spin-coating. A part of the tape was then removed prior to ALD to reveal a TFB-free area. To increase the surface sensitivity and reduce charging of the SEM and EDS samples, a 1 nm layer of Pt or a 5 nm layer of Au was sputtered on top.

### EQE_EL_ Measurements

EQE_EL_ measurements were performed using two different setups. The majority of measurements were performed using a calibrated photodiode (Thorlabs FDS 1010) directly above the LED in a N_2_ glovebox. This measurement assumed a Lambertian emission profile, the view factor between the photodiode and the LED, and an electroluminescence emission spectrum measured separately using a calibrated grating spectrometer (MayaPro 2000) to calculate the total number of photons emitted. This measurement setup is described in further detail in ref (64).

In addition, some measurements were performed in an integrating sphere with the sample placed in an air-free holder mounted to the side of the sphere. The emission spectrum and intensity were simultaneously measured by an OceanOptics QEPro calibrated grating spectrometer. This setup was used to cross-reference values with the photodiode setup and to measure the spectrum as a function of bias and time.

Both setups measured current density–voltage characteristics using a 2400 series Keithley source-measure unit.

To account for the dynamic nature of PeLED aging, care was taken to measure all samples on comparable time scales since fabrication of the device. Because the PeLEDs age rapidly under bias, each pixel was scanned repeatedly until it had reached its peak efficiency. Only the peak efficiency of each pixel was used to compile the box plots in this publication. This way, differences in efficiency due to difference in aging time could not bias the results.

### Unipolar Device Measurements

The unipolar devices were measured with the same setup used for measuring EQE_EL_. Pixels were scanned from *X* to −*X*, where *X* is 3, 5, or 10 V. The first scan on each pixel was used to compile the 3 V snapshot plotted in the paper.

### PLQY Measurements

PLQY measurements were carried out in an integrating sphere with encapsulated samples, fabricated as described above, following the procedure of de Mello et al.^68^ Lasers with a wavelength of 450 nm were used for excitation; this wavelength was carefully chosen because it is just below the absorption onset of TFB. Two different laser diodes were used: ThorLabs CPS450 and ThorLabs LP1600MM. In each case the intensity on the sample was 47.1 mW/cm^2^. The spectrum and intensity of the photoluminescence were measured by an OceanOptics QEPro calibrated grating spectrometer. For each sample, three measurements were taken, each in a different location on the sample. Care was taken each time to ensure the laser spot hit a location within the encapsulated area of the sample. The average PLQYs of the three measurements were then calculated per sample and used as one data point for the analysis and plots in the paper.

### Absorbance Measurements

Absorbance of a perovskite film spun directly on a microscope glass slide was measured on a Cary 300 Bio UV–visible spectrometer.

### Profilometry Measurements

Profilometry was carried out on a Veeco Dektak 150 profilometer. A razor blade was used to scrape a line in the film, and the profile of the line was measured.

### Ellipsometry Measurements

Ellipsometry was carried out on a J.A. Woollam RC2 spectroscopic ellipsometer. Samples were measured at three to five angles between 50 and 75°. In each case, a clean substrate was measured first and used as a foundation for the model of the samples. Fitting was performed in CompleteEASE. The TFB material was fitted by a Cauchy fit in the transparent region, which was then extrapolated into the absorbing region by a B-spline. This gave an excellent fitting result. A Cauchy fit in the transparent region was also used to determine the thickness of SnO_2_, again with an excellent fit. For TFB–Al_2_O_3_ layers, several models were tried, including Cauchy fits in the transparent region, planar bilayers (TFB then Al_2_O_3_), and Bruggeman effective medium approximations (EMAs) with TFB and Al_2_O_3_. All methods gave very similar results for the thickness. In the end, a Cauchy fit in the transparent region was used to get the data presented in Figure S17 in the Supporting Information.

### XRR Measurements

X-ray reflectivity (XRR) data was acquired with a Rigaku SmartLab diffractometer. This comprised a 1.8 kW (Cu Kα, 1.5406 Å) source with parallel beam optics with a 10 mm length limiting slit and 2.5° Soller slits. The sample were measured from 0 to 4° with a step size of 0.01° in continuous mode. X-ray scattering was collected with a HyPix-3000 2D hybrid pixel-array detector at a sample-to-detector distance of 300 mm. Data was processed using GenX ver. 3.4.11,^69^ and the film thickness was fitted as a single-layer polymer layer on the substrate with density and roughness parameters of the substrate and film allowed to float.

### SEM and EDS Measurements

Scanning electron microscopy was performed on a FEI Quanta 600 FEG microscope using a secondary electron mode. For top-down measurements, the accelerating voltage was kept as low as possible to reduce penetration depth and enhance surface sensitivity of the low-density polymer surface. EDS was performed in the same SEM, using an x-act detector from Oxford Instruments. EDS was done at 10 keV.

### AFM Measurements

Atomic force microscopy measurements were carried out using an Asylum MFP3D microscope in AC (tapping) mode in air. Olympus AC240-TS silicon tips were used for topography measurements.

## Supporting Information Available

Emission spectrum of the LEDs, data and procedure for determining the thickness of TFB layers, complementary EQE(*J*) and *J*(*V*) plots for device data presented in the main text, SEM micrographs, device schematics of unipolar devices, morphology considerations of ITO and ITO/SnO_2_ contacts, current density statistics of devices with various numbers of ALD cycles, AFM micrographs, layer RMS roughness determined by AFM, EQE(*J*) plot of the champion device, EQE(time) plot showing stability with and without an Al_2_O_3_ interlayer, skeletal diagram of the TFB molecular structure, EDS maps of Al_2_O_3_ on TFB and ITO, and thickness data of TFB-Al_2_O_3_ mixed layers (PDF)
