# Supplementary material for: Reducing Nonradiative Losses in Perovskite LEDs through Atomic Layer Deposition of Al2O3 on the Hole-Injection Contact
Source: ACS Nano. 2023 Feb 15;17(4):3289–300. doi: 10.1021/acsnano.2c04786 (PMC9979650; doi:10.1021/acsnano.2c04786)
Supplement: Supplementary file 1 — nn2c04786_si_001.pdf [file nn2c04786_si_001.pdf]

Supplementary Information:

Reducing Nonradiative Losses in Perovskite LEDs Through Atomic Layer Deposition of Al_2O_3 on the Hole-injection Contact

Emil G. Dyrvik, Jonathan H. Warby, Melissa M. McCarthy, Alexandra J. Ramadan, Karl-Augustin Zaininger, Andreas E. Lauritzen, Suhas Mahesh, Robert A. Taylor, Henry J. Snaith.*

Clarendon Laboratory, Department of Physics, University of Oxford, Oxford OX1 3PU, U.K.

2. **Figure S1.** Absorbance and PL spectrum of an isolated perovskite film on glass, EL spectrum from an LED.
3. **Supplementary Note 1.** On the determination of TFB thicknesses
4. **Figure S2.** Thickness of the deposited TFB layer on various substrates, measured using profilometry, ellipsometry and XRR.
5. **Supplementary Table 1.** Thicknesses of TFB layers.
6. **Figure S3.** EQE as function of current density for devices with varying TFB thickness and without an Al_2O_3 interlayer.
7. **Figure S4.** SEM micrographs of the ITO glass substrate and the ITO glass substrate with TFB and TFB-Al_2O_3 layers, respectively.
8. **Figure S5.** Device schematic of the unipolar, electron-only devices with and without the ALD-Al_2O_3 interlayer.
9. **Figure S6.** JV-characteristics of one representative measurement each on unipolar electron-only devices.
10. **Supplementary Note 2.** Morphology considerations of ITO and ITO/SnO_2 contacts.
11. **Figure S7.** SEM micrographs of ITO and ITO/SnO_2 contacts.
12. **Supplementary Note 3.** Implications of the current density data in Figure S7.
13. **Figure S8**. The current density at 3.5 V during JV-scans of LEDs made with 11 nm TFB and various numbers of Al2O3 ALD cycles.
14. **Figure S9.** AFM micrographs of thin films with and without the ALD-Al_2O_3 layer.
15. **Figure S10.** AFM micrographs of the perovskite films on various substrates.
16. **Supplementary Table 2.** Root mean square roughness of layers determined by AFM.
17. **Figure S11.** Champion device measured in our lab. Device structure ITO/TFB/Al_2O_3/LiF/Perovskite/TPBi/LiF/Al. TFB 5 mg/ml, 10 cycles of ALD.
18. **Figure S12.** JVL plot of devices with varying thickness of TFB with an Al_2O_3 interlayer (10 cycles of ALD).
19. **Figure S13.** EQE as function of current density for devices with varying thickness of TFB with an Al_2O_3 interlayer.
20. **Figure S14.** EQE as function of time for devices with and without the interlayer.
21. **Figure S15.** Skeletal diagram of poly(9,9-dioctylfluorene-alt-N-(4-sec-butylphenyl)-diphenylamine) (TFB).

22. **Figure S16.** EDS map of TFB-covered versus TFB-free areas on an ITO sample.
23. **Figure S17.** Thickness and increase in thickness as function of number of ALD-Al_2O_3 cycle exposures.
24. **Figure S18.** Energy band diagram schematics of the various interfaces with the perovskite emitter.

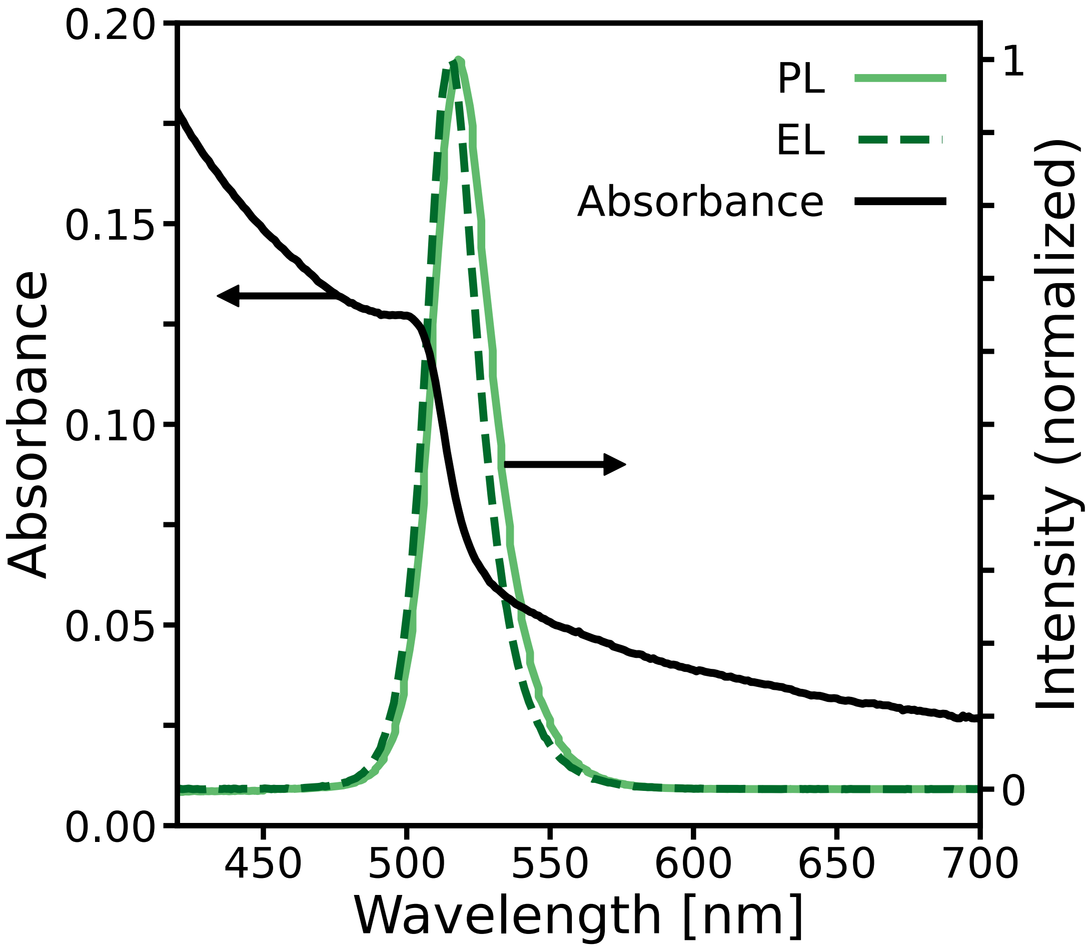

Figure S1. Absorbance and PL spectrum of an isolated perovskite film on glass, EL spectrum from an LED.

Supplementary Note 1

We measure the thickness of TFB layers spin-coated from solutions of different concentration, and on various substrates, by means of profilometry, ellipsometry and x-ray reflectivity (XRR). The results are plotted in Figure S2. Using the data in Figure S2, we take the mean of the measured values to determine the thickness of TFB layers from solutions with 0.5, 2, 5 and 10 mg/ml TFB in chlorobenzene. The resulting mean thicknesses and standard deviations are given in Supplementary Table S1. From these mean values, we use linear interpolation to determine the thickness of layers spun with solutions of 1, 3 and 8 mg/ml. These were interpolated between their nearest values, e.g. 1 mg/ml was determined by linear interpolation between 0.5 and 2 mg/ml.

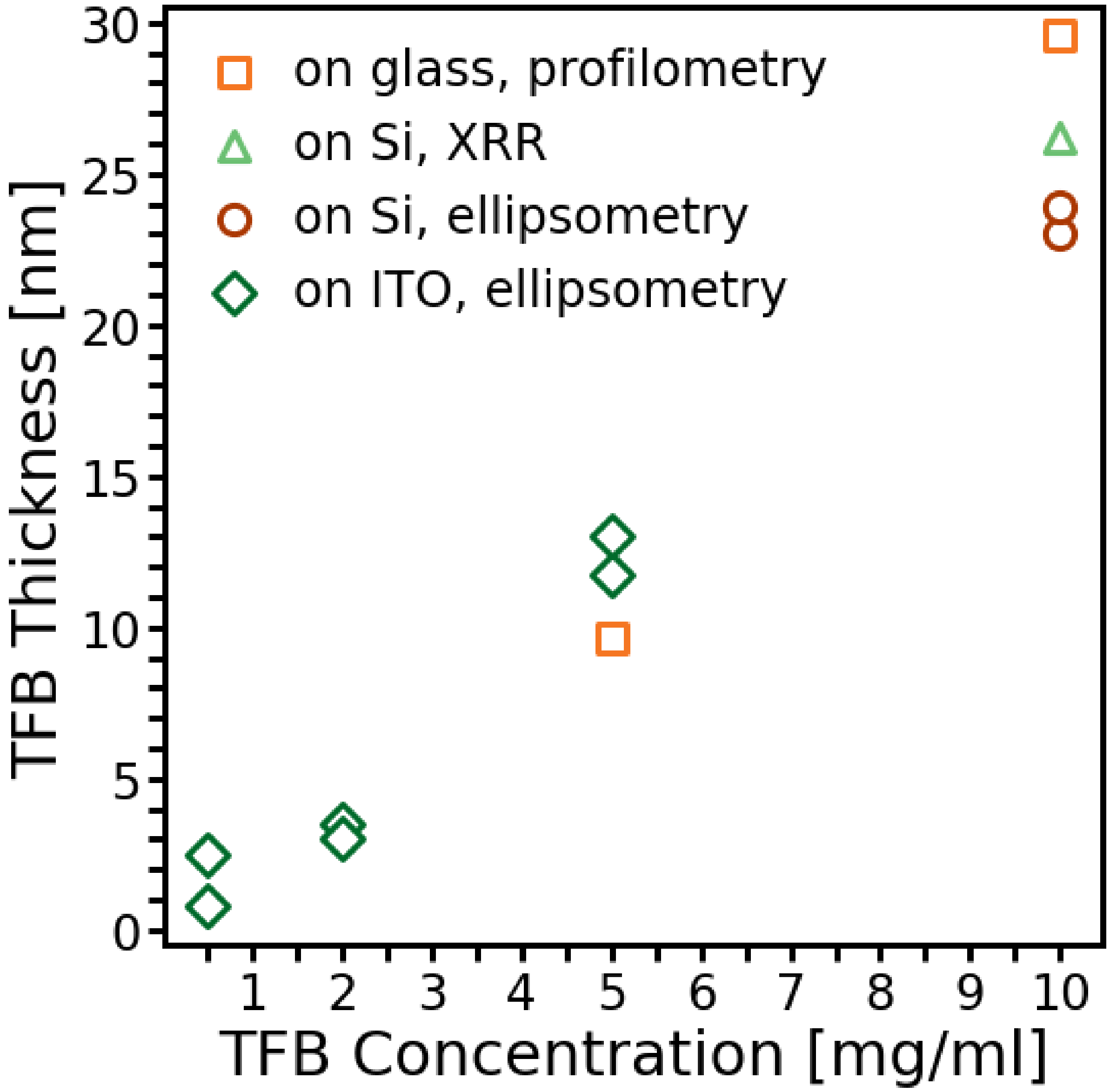

Figure S2. Thickness of the deposited TFB layer on various substrates, measured using profilometry, ellipsometry and XRR.

Supplementary Table 1. Thicknesses of TFB layers.

TFB Concentration [mg/ml]	Method	Thickness [nm]	Std.dev. [nm]
0.5	Mean	1.63	1.19
1.0	Linear interpolation	2.17	-
2.0	Mean	3.25	0.35
3.0	Linear interpolation	6.00	-
5.0	Mean	11.5	1.7
8.0	Linear interpolation	20.0	-
10.0	Mean	25.7	2.9

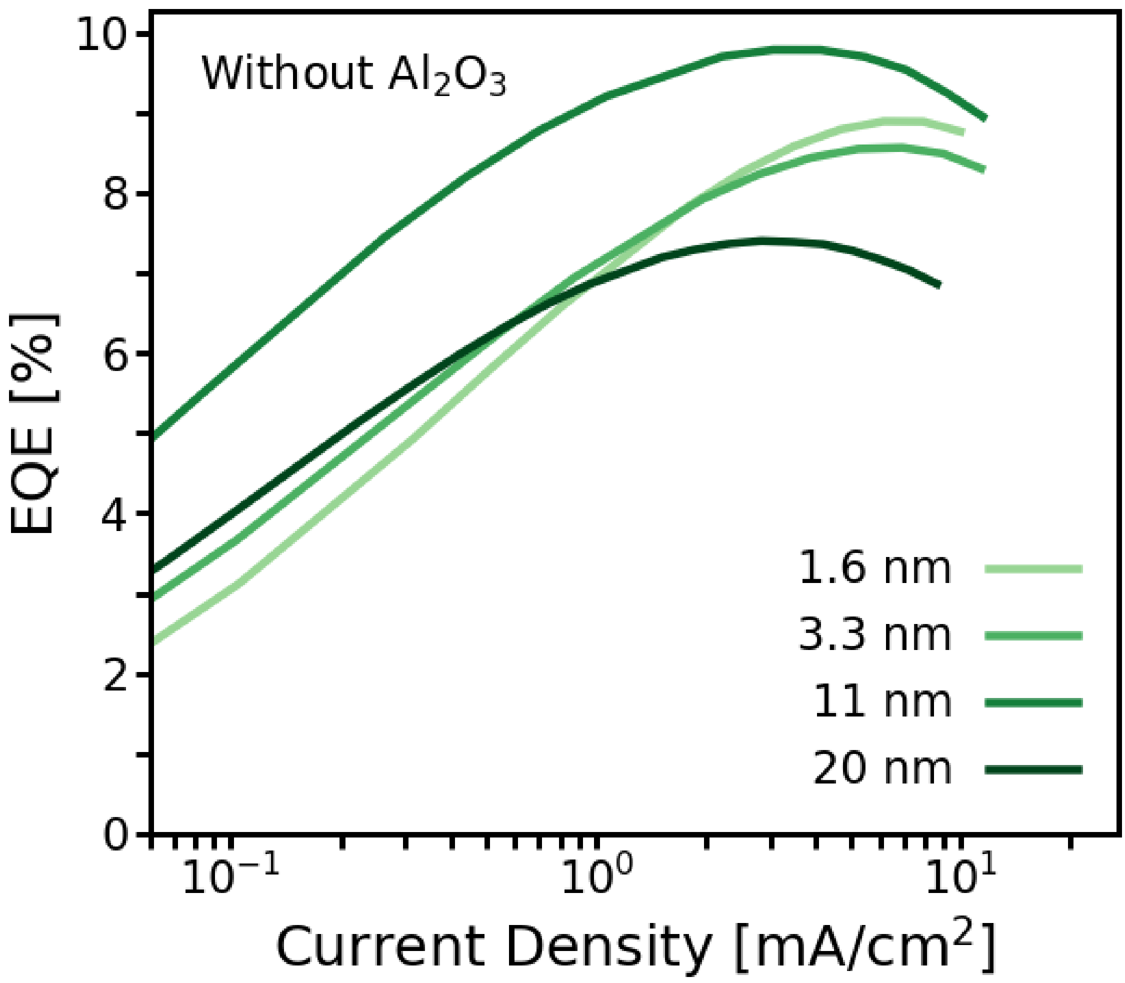

Figure S3. EQE as function of current density for devices with varying TFB thickness and without an Al_2O_3 interlayer.

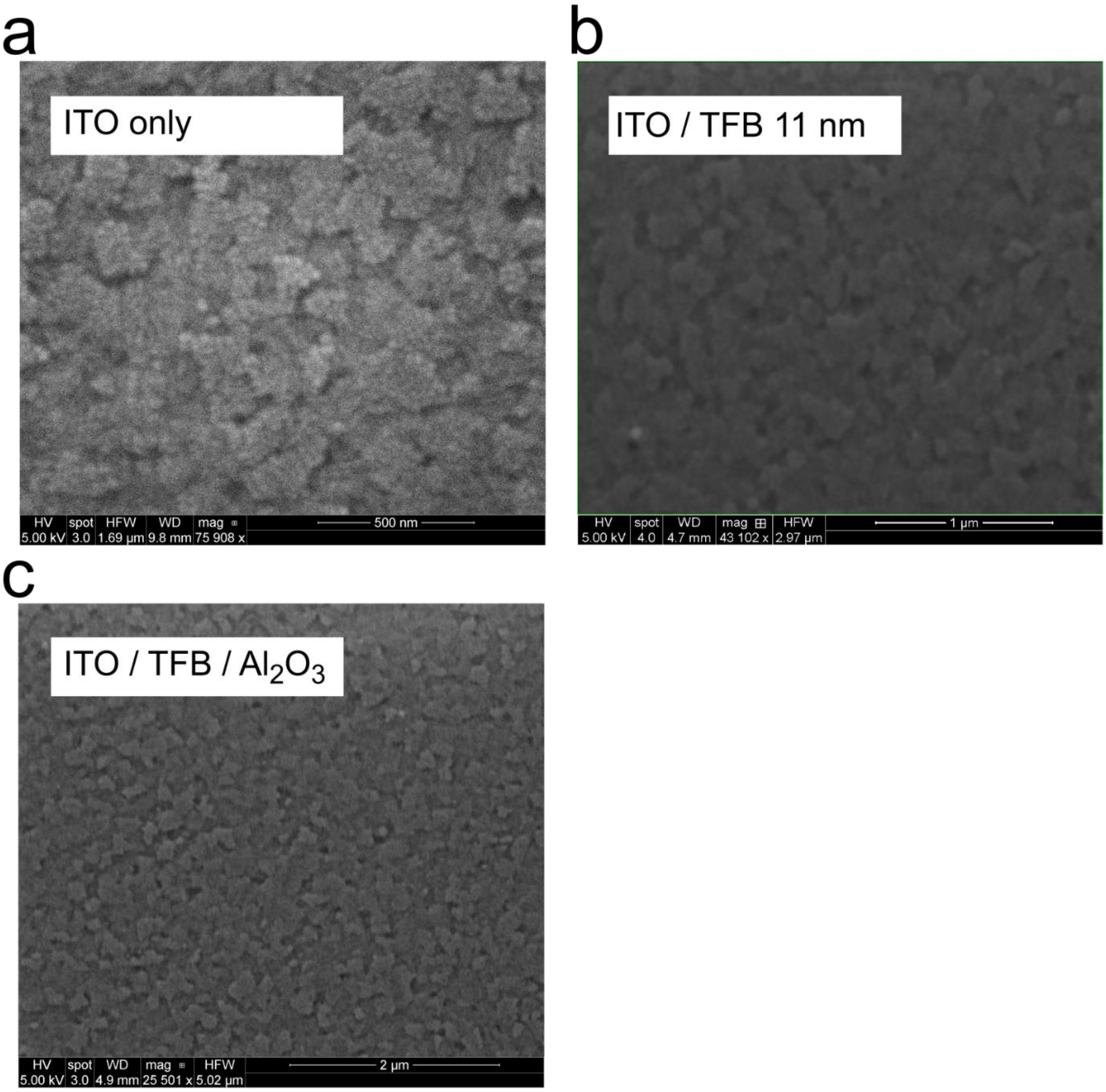

Figure S4. SEM micrographs of the ITO substrate and the ITO substrate with TFB and TFB-Al_2O_3 layers, respectively.

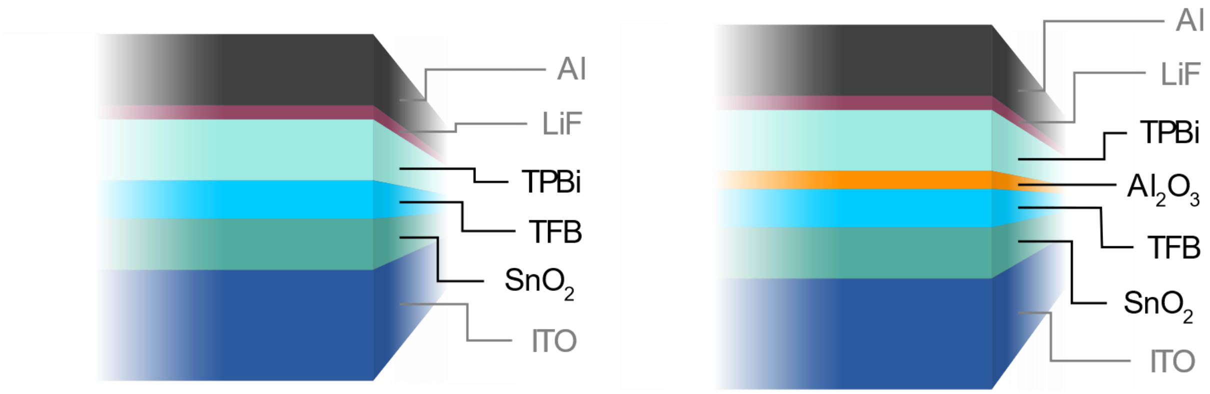

Figure S5. Device schematic of the unipolar, electron-only devices with and without the ALD-Al_2O_3 interlayer.

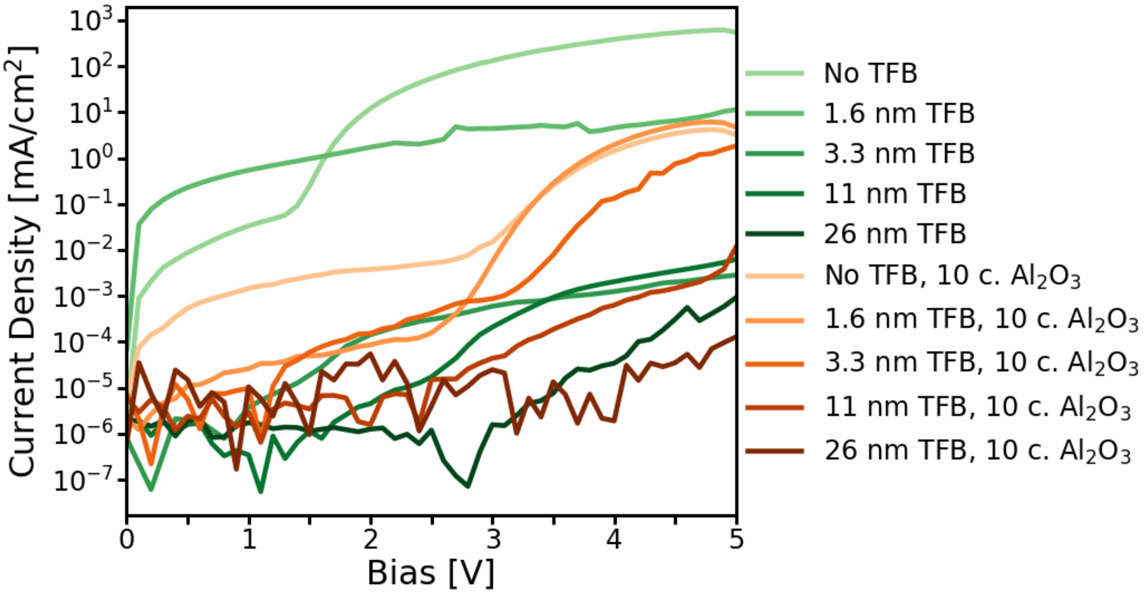

Figure S6. JV-characteristics of one representative measurement each on unipolar electron-only devices. The modulus of the current density is plotted because of some negative value data near the noise limit of the instrument that cannot be displayed on a log plot.

Supplementary Note 2

We have inspected ITO substrates with and without SnO_2 by SEM (Figure S7). The top-down micrographs (a and b) do not display any notable difference in the surface morphology. However, we note that due to the penetration depth of the SEM beam it is likely we probe deep into the ITO layer.

We also investigated an ITO-SnO_2 sample cross-section, Figure S7 (c-f). The SnO_2 layer has partially flaked off the substrate closest to the cleavage plane when the sample was cleaved for the cross-section, revealing both the ITO surface and a thin layer of SnO_2 further into the sample. The SnO2 layer is visible as one or more layers of nanoparticles over the surface. The coating seems conformal, except for where the layer has flaked off during handling leaving debris on the surface. The micrographs suggest that the SnO_2 layer is somewhat rougher than the ITO substrate.

With ellipsometry of a SnO_2 layer on Si, assuming perfectly flat layers, we estimate the SnO_2 layer thickness to be 15 nm. This corresponds well with the observations from SEM, considering the surface roughness of the real layers.

Despite the differences in morphology of the SnO_2 and ITO layers, the data from unipolar electron-only devices still provides useful information. Figure 2e shows that the incorporation of an ALD-Al_2O_3 interlayer provides a larger reduction in electron leakage current for thinner TFB layers than for thicker TFB layers. The roughness of the underlying substrate may affect the overall degree to which the leakage current is reduced for the thinner layers, but we expect the same trend to occur. The fact that we observe an optimum LED performance with an 11 nm TFB (Fig 1b) without the Al_2O_3 interlayer while we observe an optimum with 3.3 nm TFB when using a 10 cycle Al_2O_3 interlayer (Fig 4d) reinforces the observation that the effect of supressing leakage current is greater for thinner TFB layers.

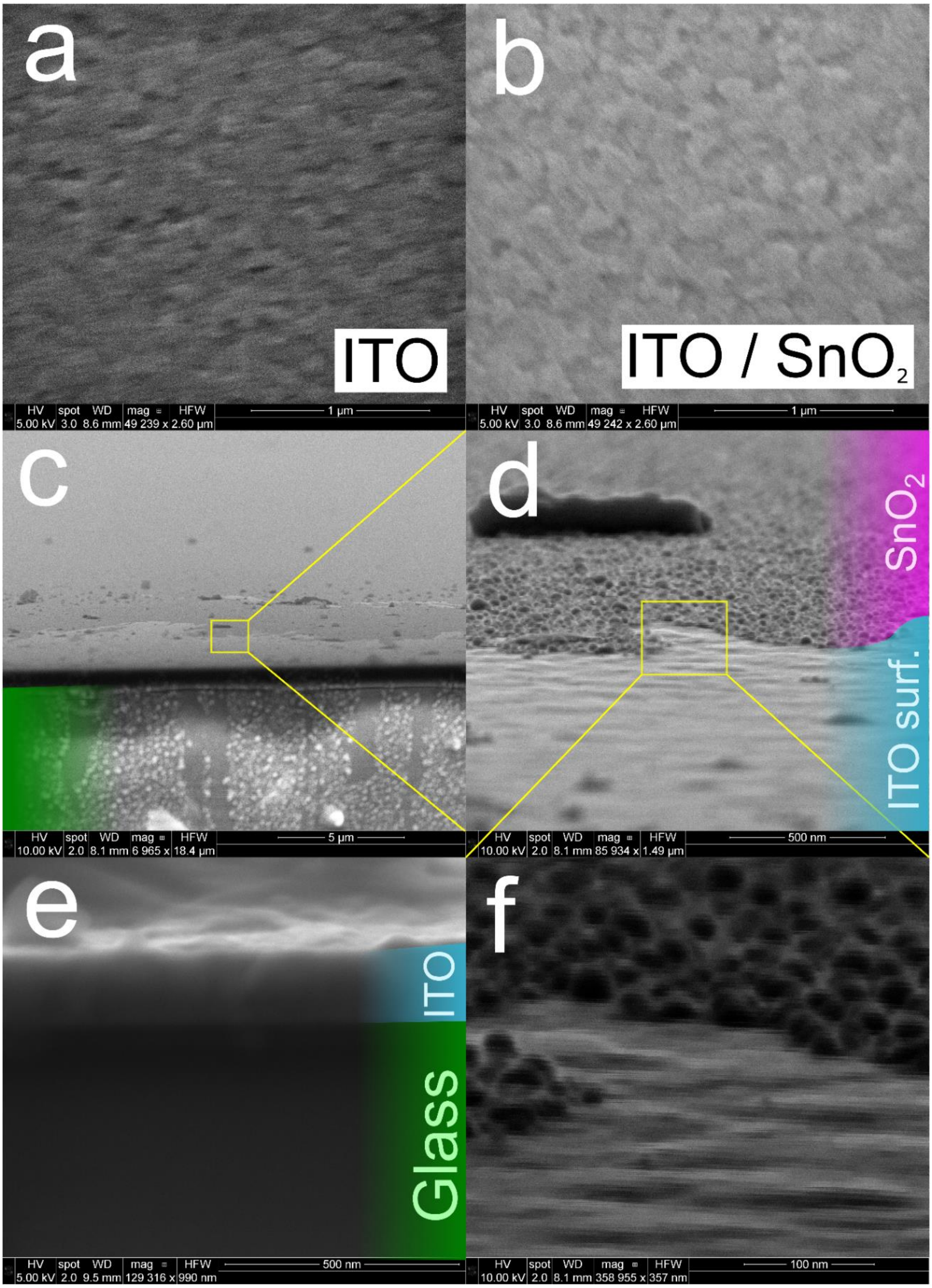

Figure S7. SEM micrographs of ITO and ITO-SnO_2 samples. (a) top-down micrographs of the ITO substrate; (b) top-down of ITO-SnO_2 contact; (c) cross-section of ITO-SnO2 sample showing (from bottom to top) the glass cross-section, the ITO surface close to the cleavage where the SnO_2 has flaked off, and the SnO_2 surface; (d) zoomed area from cross-section in (c); (e) the ITO cross-section with a SnO2-free surface next to the cleavage plane; (f) zoomed area from (d).

Supplementary Note 3

In Supplementary Figure S8 we plot the current density at 3.5 V as function of ALD cycles. The variation in current density is large for each sample type, but larger for 0 and 5 ALD cycles, and there is a large overlap between the boxes in the boxplot. Furthermore, the lower extreme is approximately the same for all sample types, whereas there are larger differences in the upper extreme. This is what would be expected for a reduction in the probability of active pinholes in the device. We also see that there is virtually no difference in the current density for samples with 10 versus 20 cycles of ALD, further indicating that the insulating effect of Al_2O_3 does not decrease the current density except for a reduction in nonradiative current.

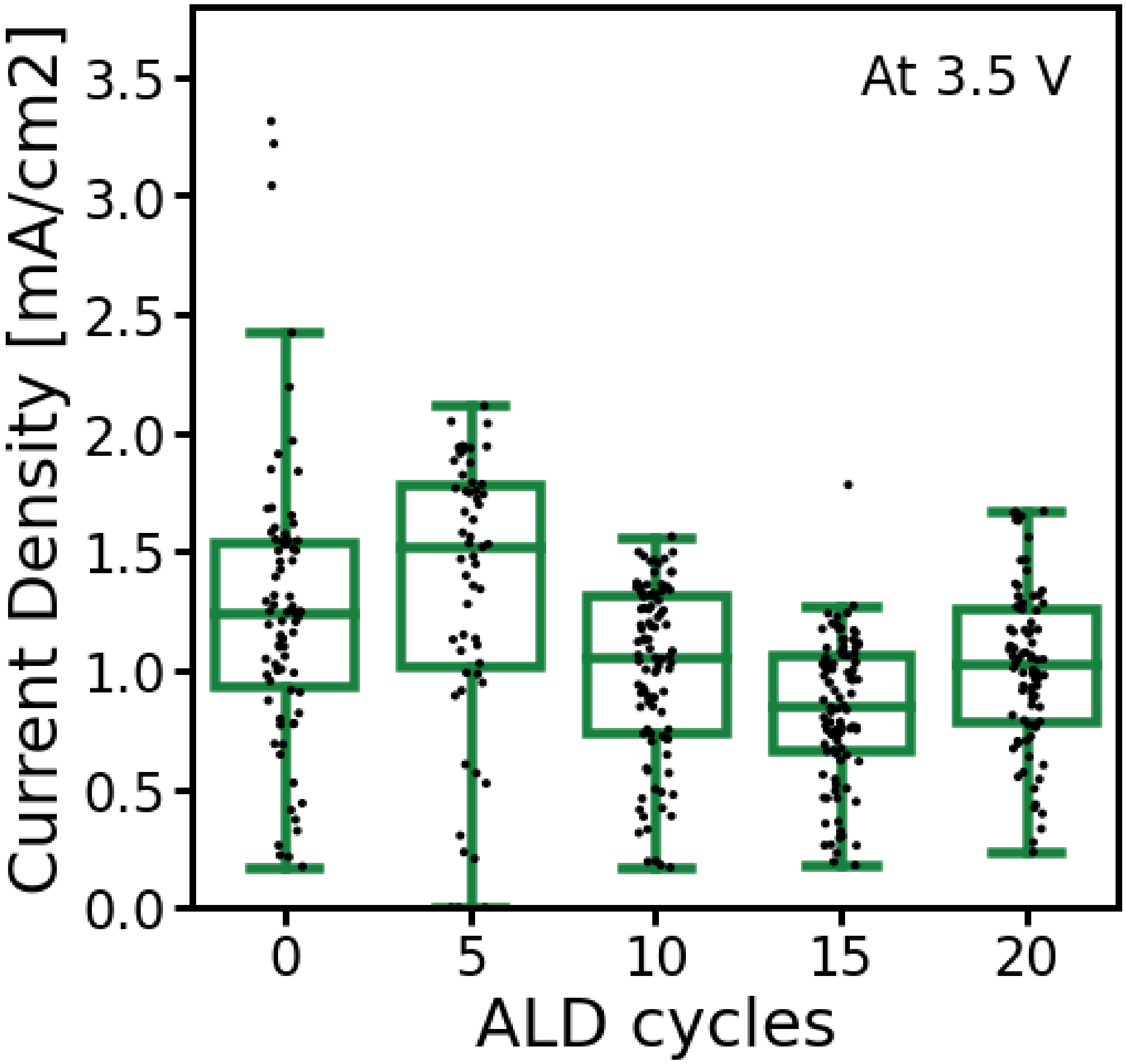

Figure S8. The current density at 3.5 V during JV-scans of LEDs made with 11 nm TFB and various numbers of Al_2O_3 ALD cycles. The data comprises 413 scans across 42 different samples.

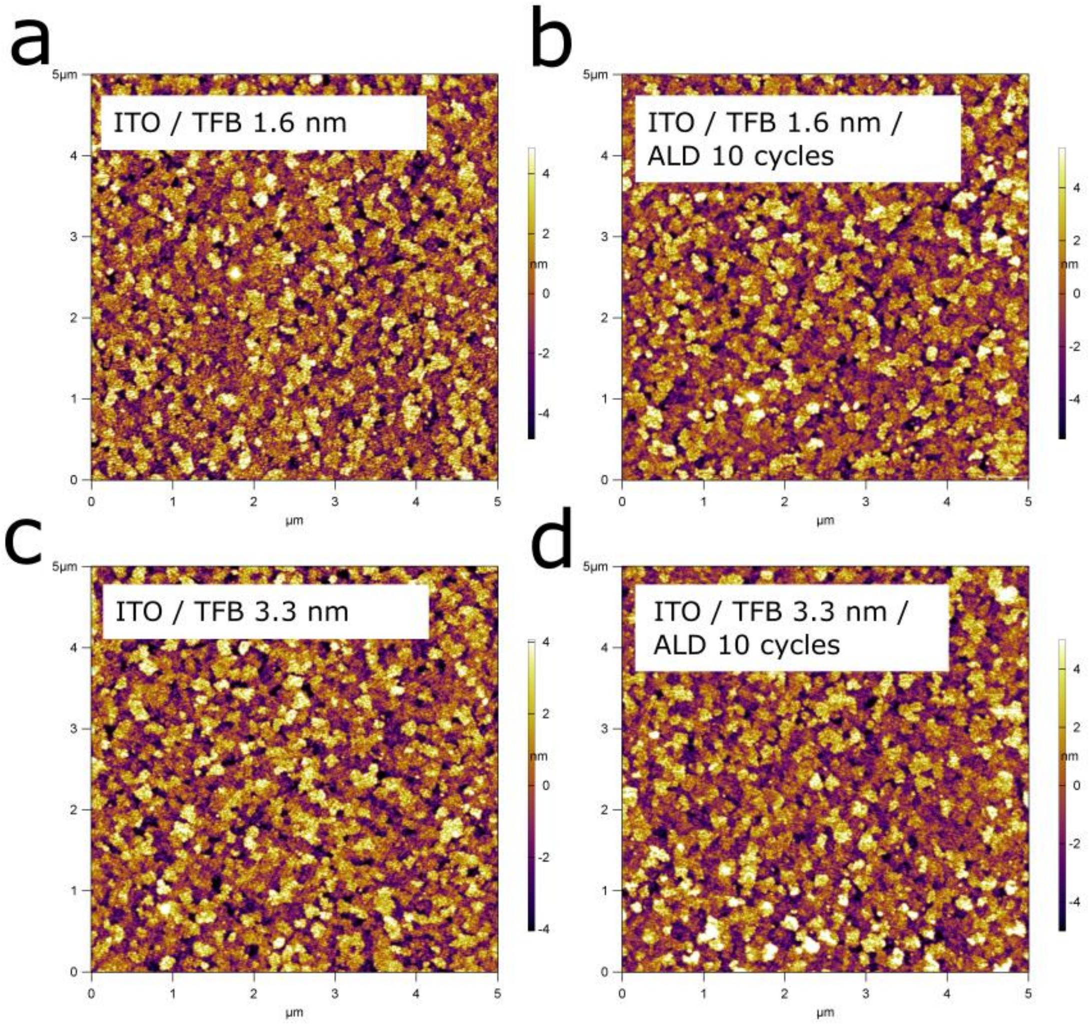

Figure S9. AFM micrographs of thin films with and without the ALD-Al_2O_3 layer.

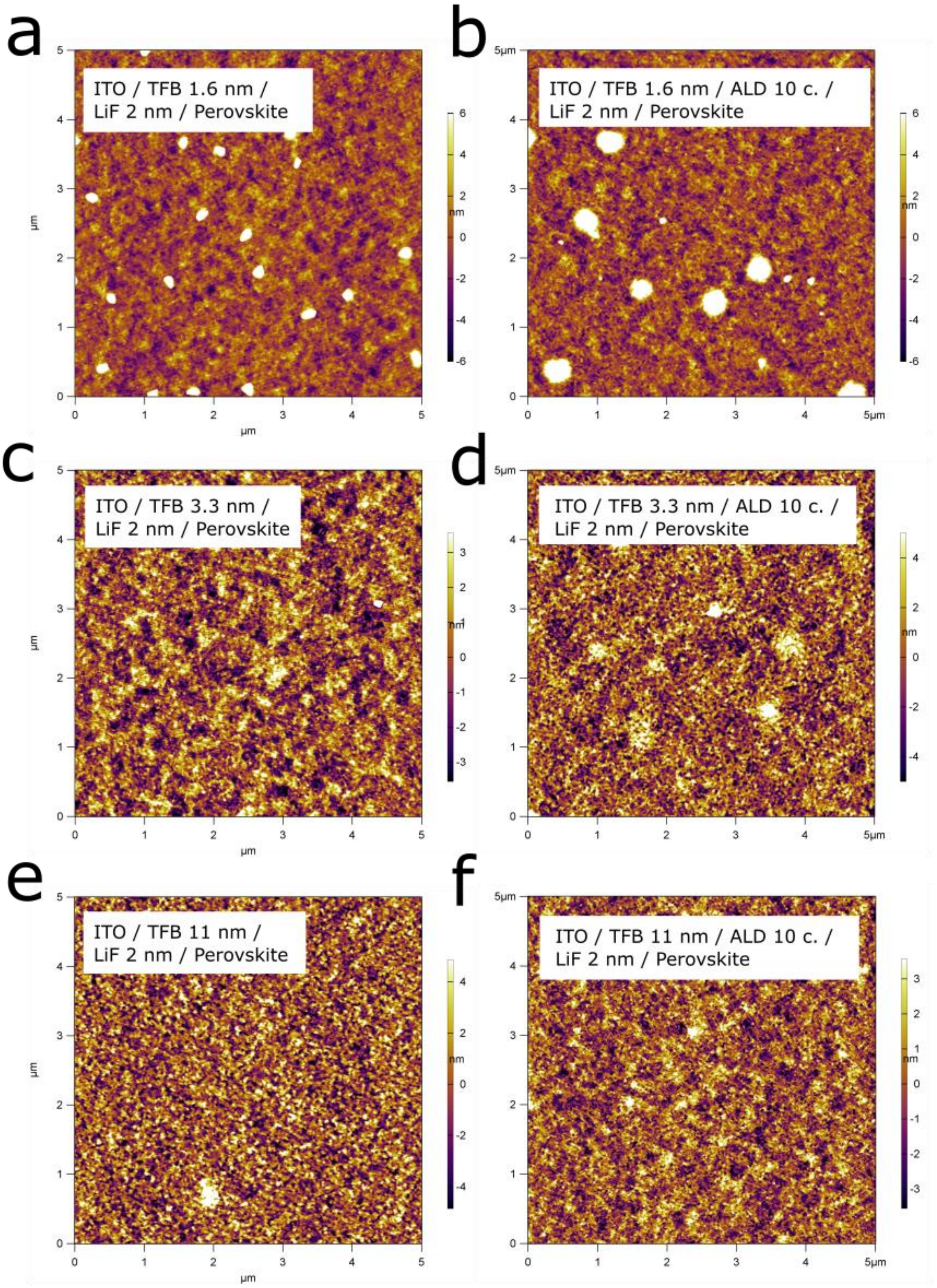

Figure S10. AFM micrographs of the perovskite films on various substrates.

Supplementary Table 2. Root mean square roughness of layers determined by AFM.

Stack	Roughness [nm]
ITO	3.3
ITO / TFB 1.6 nm	2.7
ITO / TFB 1.6 nm / Al2O3 10 c.	2.7
ITO / TFB 1.6 nm / Al2O3 10 c. / LiF 2 nm	2.3
ITO / TFB 1.6 nm / LiF 2 nm / perovskite	3.1
ITO / TFB 1.6 nm / Al2O3 10 c. / LiF 2 nm / perovskite	3.1
ITO / TFB 3.3 nm	2.3
ITO / TFB 3.3 nm / Al2O3 10 c.	2.5
ITO / TFB 3.3 nm / LiF 2 nm / perovskite	1.8
ITO / TFB 3.3 nm / Al2O3 10 c. / LiF 2 nm / perovskite	2.6
ITO / TFB 11 nm	1.7
ITO / TFB 11 nm / LiF 2 nm / perovskite	2.4
ITO / TFB 11 nm / Al2O3 10 c. / LiF 2 nm / perovskite	1.7
ITO / TFB 11 nm / Al2O3 10 c. / LiF 2 nm / perovskite, spot 2 (not pictured)	1.3

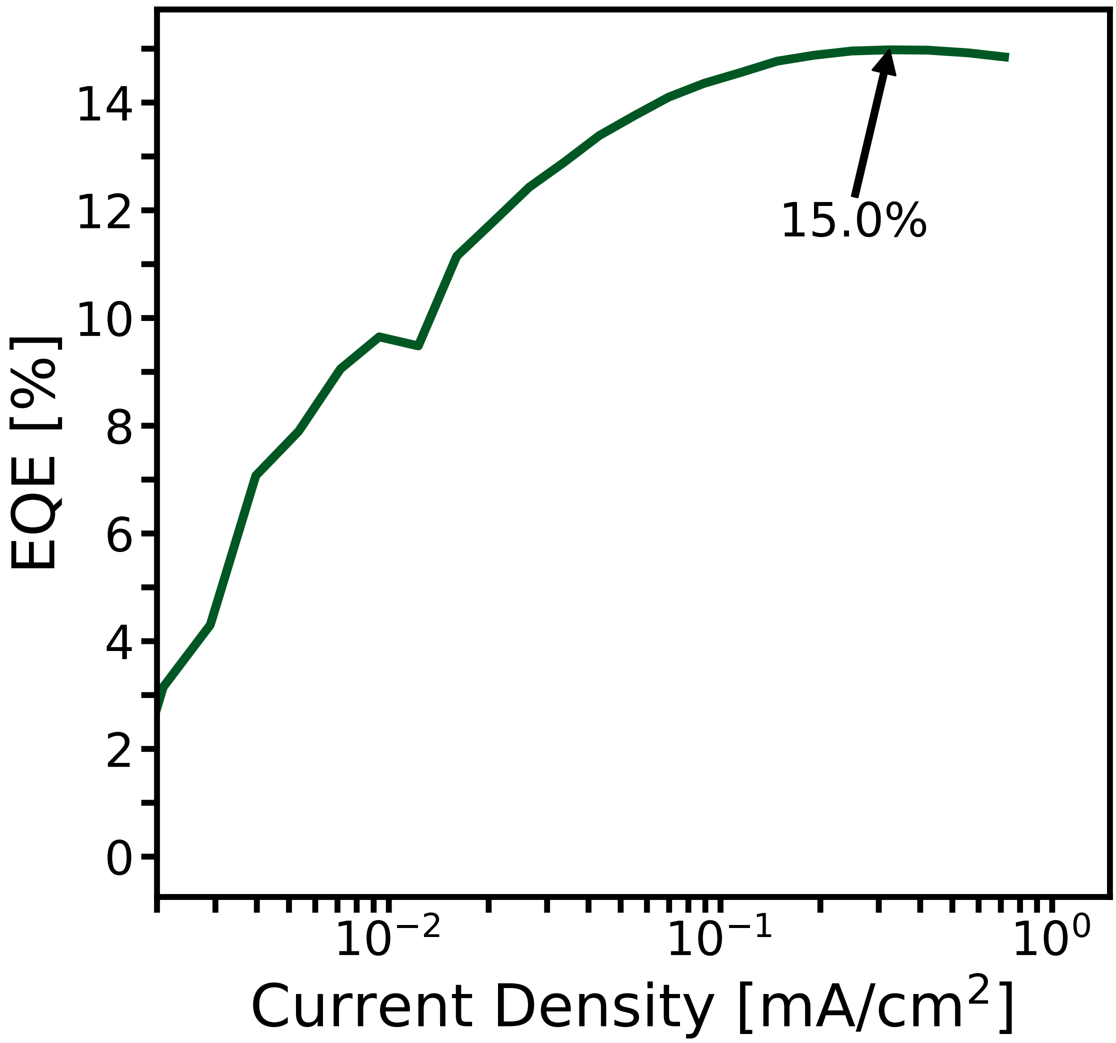

Figure S11. Champion device measured in our lab. Device structure ITO/TFB/Al_2O_3/LiF/Perovskite/TPBi/LiF/Al. TFB 11 nm, 10 cycles of ALD.

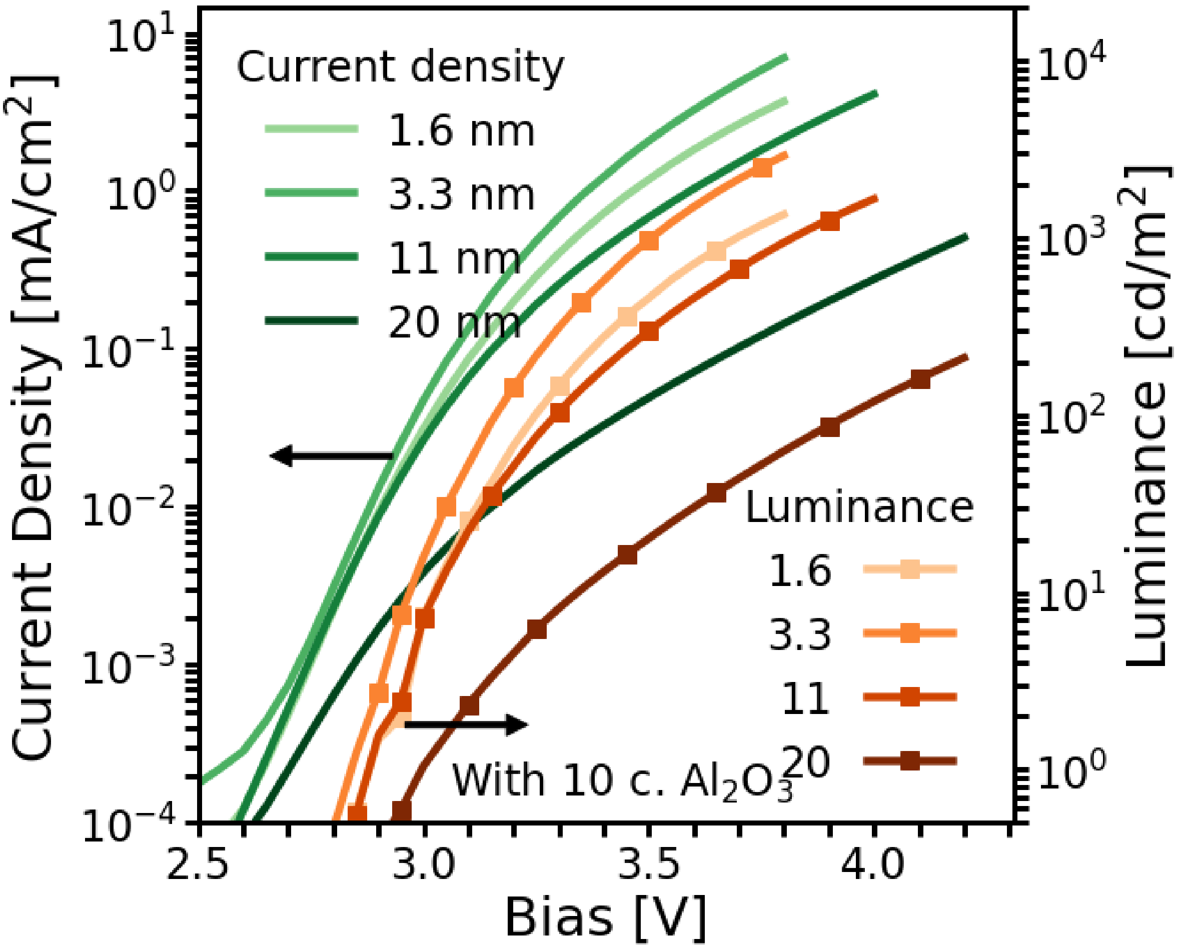

Figure S12. JVL plot of devices with varying thickness of TFB with an Al_2O_3 interlayer (10 cycles of ALD).

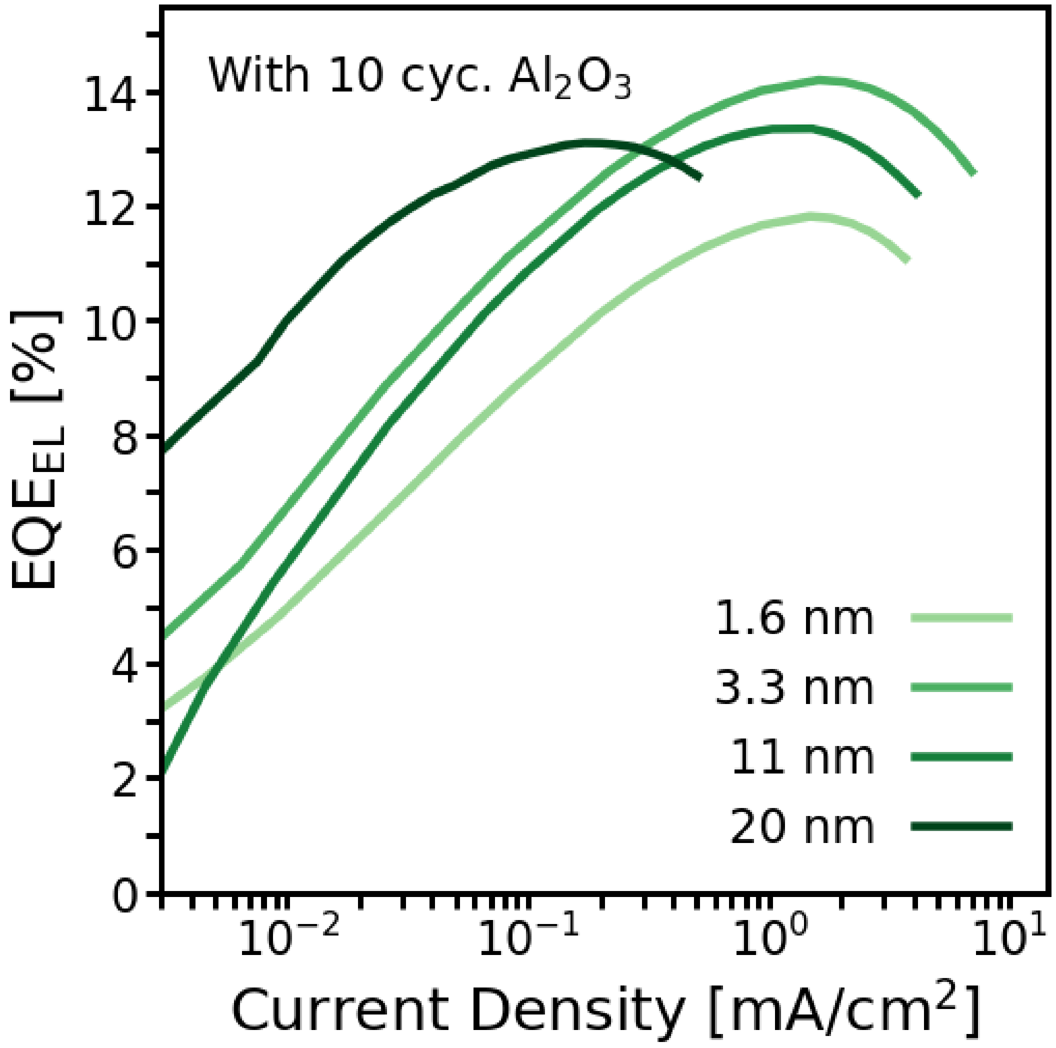

Figure S13. EQE as function of current density for devices with varying thickness of TFB with an Al_2O_3 interlayer.

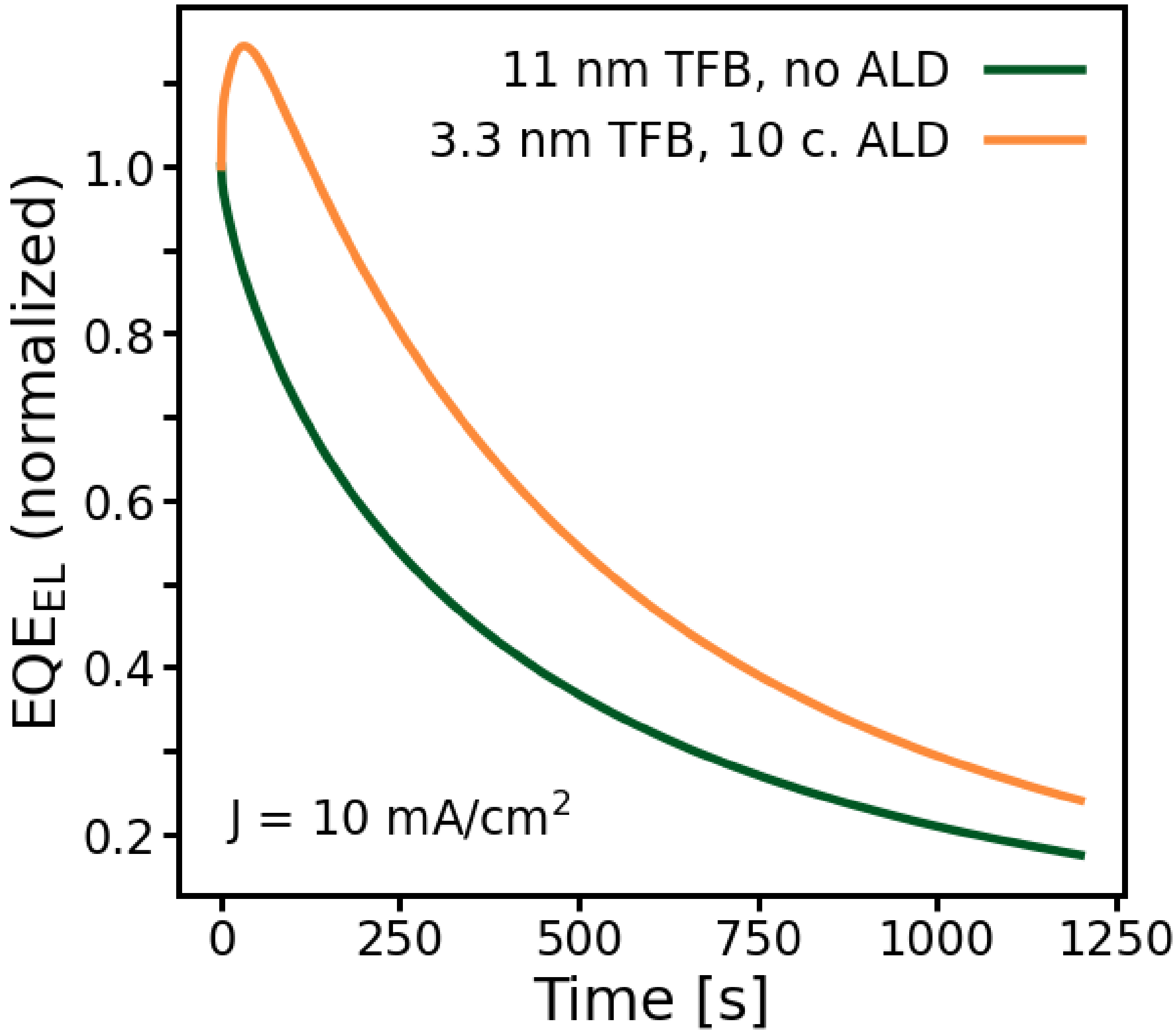

Figure S14. EQE as function of time for devices with and without the interlayer. Except for a change in the initial response, the decay rate is largely similar.

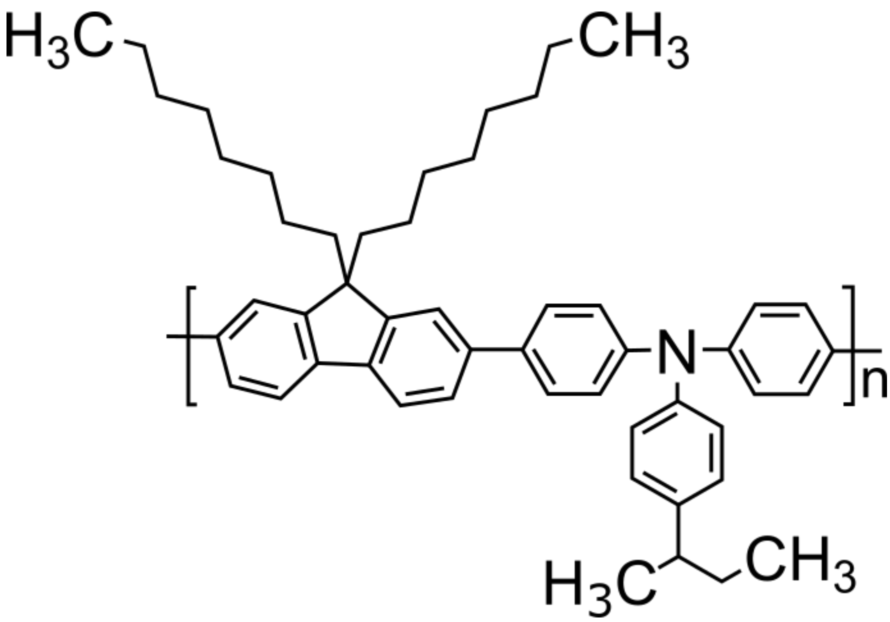

Figure S15. Skeletal diagram of poly(9,9-dioctylfluorene-alt-N-(4-sec-butylphenyl)-diphenylamine) (TFB).

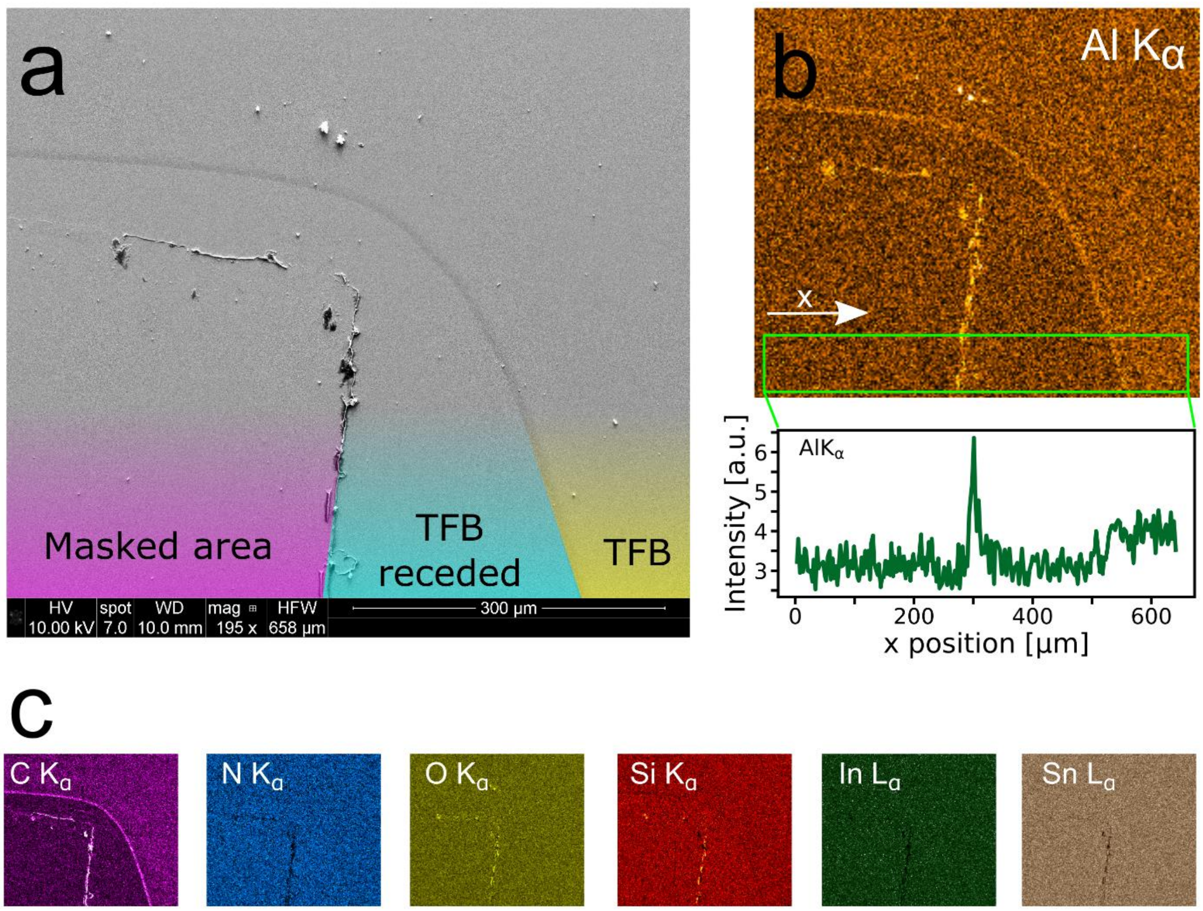

Figure S16. (a) SEM secondary electron micrograph of the location on that was EDS mapped. The sample was an ITO substrate with 11 nm TFB and 20 cycles ALD-Al_2O_3, where parts of the sample was masked by polyimide tape during TFB spin-coating. The color coding indicates: (magenta) the area that was masked and is therefore TFB-free; (cyan) an area around the masking tape where the TFB had receded after spin-coating; (yellow) a TFB-covered area. (b) EDS map of the Al K_α counts over the area, with a plot of the average intensity along x within the indicated rectangle. (c) Maps of EDS intensities of other elements over the same area.

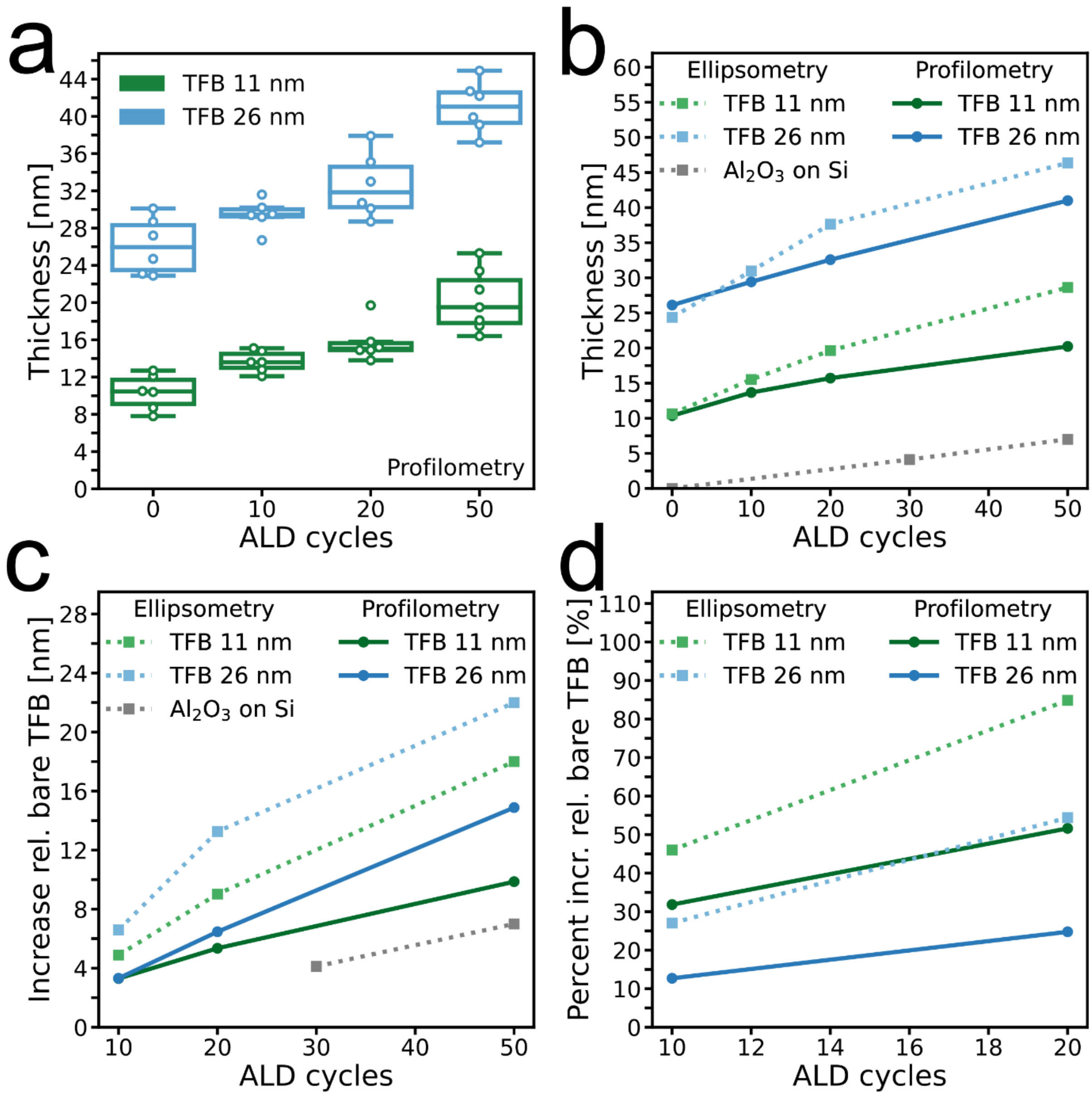

Figure S17. (a) Boxplots of profilometry measurements of 6-7 samples per physical sample, showing the distribution of results. (b) Thickness of samples as function of ALD-Al_2O_3 cycles exposed to. Thicknesses were determined by profilometry (average values) and ellipsometry. ALD-Al_2O_3 on Si shows close to expected linear growth of 1.1 Å per cycle. (c) The thickness increase for each sample relative to the 0 ALD samples. (d) The percent increase in thickness of samples exposed to 10 or 20 ALD cycles relative to 0 cycles.

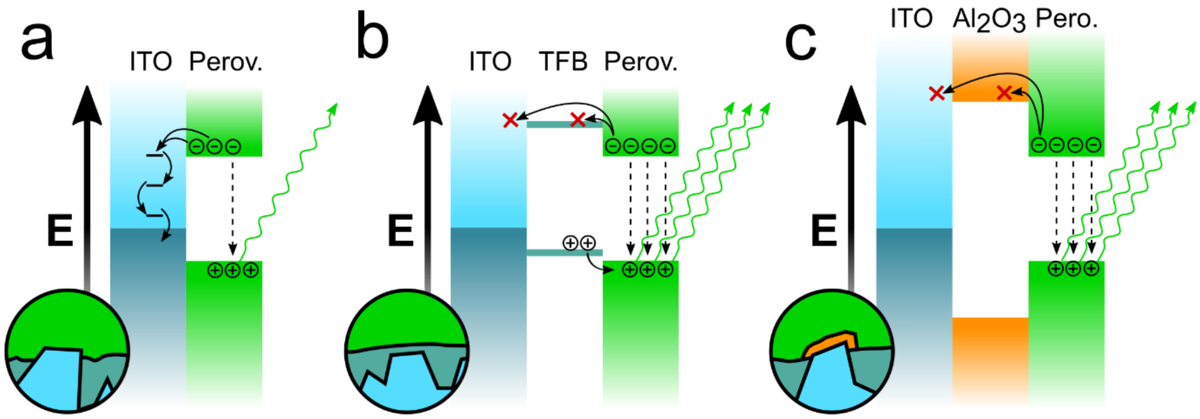

Figure S18. (a) Energy band schematic displaying efficient hole injection and electron-blocking behavior with TFB; (b) electron leakage at the ITO–perovskite interface when the ITO is left exposed; (c) efficient electron blocking with Al_2O_3 covering the exposed ITO. Not to scale.
